# Robust circular cluster-based statistics for respiration-brain coupling

**DOI:** 10.1371/journal.pcbi.1014672

**Published:** 2026-09-03

**Authors:** Teresa Berther, Elio Balestrieri, Martina Saltafossi, Laura Bock Paulsen, Lau M. Andersen, Daniel S. Kluger

**Affiliations:** 1 Institute for Biomagnetism and Biosignal Analysis, University of Münster, Münster, Germany; 2 Otto Creutzfeldt Center for Cognitive and Behavioral Neuroscience, University of Münster, Münster, Germany; 3 Department of Linguistics, Cognitive Science and Semiotics, Aarhus University, Aarhus, Denmark; 4 Center of Functionally Integrative Neuroscience, Aarhus University, Aarhus, Denmark; Ghent University, BELGIUM

## Abstract

The rapidly developing research field of brain-body neuroscience faces methodological challenges, as analysts continue to develop new analysis strategies in the absence of established best practices. This quest for valid methods is further complicated by the (naturally) circular data involved in the study of phase-locked effects, e.g., in respiration-brain coupling. Various available approaches for phase extraction, constructing adequate surrogate data for statistical comparison, and accounting for the circularity of respiratory data lead to poor cross-study generalisability of results. Interpretation of effects is particularly affected by the problem of multiple comparisons in phase-related inferential statistics. In this tutorial, we propose a robust pipeline for respiration phase-related analyses based on a novel circular extension of cluster-based permutation testing. We highlight and offer guidance on critical parameters in the analysis, systematically compare various approaches being used in the field today, and provide open-access software code for flexible use and future development of our proposed pipeline.

## Author summary

Conventional best-practice statistical approaches have so far been difficult to implement in cases where the underlying data are circular, for example in the analysis of phase data. Such analyses are common in the up-and-coming research field of brain-body neuroscience, which poses significant challenges. In this tutorial, we use the case of respiration to propose a robust pipeline for phase-related analyses based on a novel circular extension of cluster-based permutation testing. We highlight and offer guidance on critical parameters in the analysis, systematically compare various approaches being used in the field today, and provide open-access software code for flexible use and future development of our proposed pipeline.

## Introduction

### Particulars of analyzing brain-body interactions across organ systems

Brain-body interactions continue to attract increased attention in the field of neuroscience. Joint analyses of neural time series (with fMRI or M/EEG) and physiological rhythms like the heartbeat (~1 Hz), breathing (~0.25 Hz), or gastric signals (~0.05 Hz) have begun to unravel the manifold and complex ways in which the brain systematically and bidirectionally interacts with the rest of the body. Dedicated literature reviews [1,2] comprehensively highlight the universality of these coupling effects across modalities and have grounded brain-body coupling in conceptual frameworks of brain function [3,4]. Reaching far beyond fundamental research, the explanatory potential of centro-peripheral coupling is most easily recognised in clinical applications [5,6].

Despite (or maybe due to) its rapid development, the field of brain-body research faces methodological challenges, most prominently in conducting inferential statistics. Analyses of physiological parameters like breathing depth [7,8], pathways [9,10], or respiratory rate [11,12] can be conducted with straightforward statistical comparisons across two or more conditions (although exceptions certainly apply, e.g., in continuous or non-stationary recordings). Phase-locked analyses of physiological rhythms, on the other hand, are not only lacking such a contrast condition, but phase data is inherently circular and therefore do not allow for a convenient parameterization of the underlying distribution. Hence, statistical approaches currently being used to circumvent these difficulties are rather heterogeneous, which is why the present tutorial paper focuses on analyses of continuous phase. Since the field is lacking best practices for brain-body analyses based on continuous phase, we offer guidance on several degrees of freedom, e.g., how to properly extract phase information, whether to treat it as categorical or continuous, how to create adequate null distributions for statistical comparisons, and how to control for false positive results. Our aim is to provide a stepwise workflow which avoids common pitfalls for the benefit of overall robustness.

To start with, in order to relate neural activity and/or behavioural measures to the phase of physiological signals, phase vectors have to be reliably extracted from the raw signal. The way in which phase information is used for subsequent analyses greatly depends on the frequency and interpretability of the respective organ system. Questions involving the cardiac rhythm, for example, are commonly reduced to comparisons of neural or behavioural data during systole vs diastole, although this distinction comes with its caveats regarding the actual onset definitions of systole and diastole [13]. Since the ECG is a relatively fast signal which cannot be consciously modulated directly, phase-related analyses going beyond the widely accepted systole/diastole dichotomy are very rare. From a statistical point of view, this considerably simplifies analyses to parametric or non-parametric models. While such simplifications come with no obvious disadvantages in the specific case of cardiac-based analyses, they fall short of adequately representing physiological signals which are either slower in rhythmicity (gastric) and/or under voluntary control (respiration).

Although the gastric rhythm is equally non-accessible to voluntary modification, its use in brain-body neuroscience is entirely different from that of the ECG. Due to the very low frequency of the gut’s rhythmicity at around 20s per cycle, it is in principle well suited for phase binning approaches, i.e., partitioning the continuous phase of the gastric cycle into a more or less arbitrary number of equally distributed bins. However, it is far from trivial to functionally interpret gastric phase beyond the activity rate of pacemaker cell ensembles it is generated by, which is why such analyses are the exception rather than the rule. Instead, gastric phase is most commonly used in cumulative coupling analyses, i.e., quantifying the relationship between gastric phase and the phase or amplitude of neural responses over the entire recording [14,15]. In contrast to ECG-related analyses outlined above, this approach yields one value for metrics like the phase-locking value (for phase-phase coupling) or modulation index (for phase-amplitude coupling) per voxel or sensor. Group-level inference statistics are then conducted to determine whether the observed coupling between gastric phase and fMRI BOLD response or M/EEG activity is stronger than to be expected in a random partition of the data. To this end, a null distribution of ‘chance-level coupling’ is created by shuffling or circular shifting of the phase vector and recomputing the coupling metrics many times.

Finally, analyses of respiration-brain coupling constitute a special case for several reasons: Most prominently, respiration is the only physiological rhythm which is fully under voluntary control. Not only can respiratory rate be adjusted at will, but, e.g., breath holds during either inspiration or expiration give participants a great amount of flexibility in temporally aligning their breathing rhythm with critical events during tasks in neuroscientific studies [11,16]. As a consequence, respiratory time series are not simply ‘automatically’ repeating, but may undergo volitional breath-by-breath modulation in order to facilitate behaviour. This is why, despite its continued popularity, discretizing the respiratory signal into a mere contrast of ‘inspiration vs expiration’ does not adequately represent the rich and variable dynamics of the signal (for general critiques of discretizing continuous data, see [17,18]). This is further highlighted by consistent reports of respiration phase-dependent effects during transition times (e.g., from inspiration to expiration and vice versa [19–21]) or other specific phases [9,16,19,22–24] such as the strongest changes in airflow (i.e., the inflection point of the respiratory signal). Simply collapsing continuous respiratory phase into two discrete ‘states’ would make the investigator blind to detecting such momentary modulations and drastically reduces the sensitivity of respiration-related analyses. Rather, the full information from continuous respiratory phase should be exploited to maximise signal-to-noise ratio.

As mentioned at the outset, the field’s hesitancy to implement rigorous analyses of continuous respiratory phase may at least partly be explained by the absence of validated statistical approaches. Two things are needed: A reliable approach for continuous, artefact-free phase extraction, and inferential statistics with adequate control over false positive results and multiple comparison correction. On the one hand, widely used approaches based on the Hilbert transform are prone to produce artefacts and oftentimes do not provide satisfactory data quality during phase extraction (see Fig 2). On the other hand, while cluster-based permutation correction [21,25] has become the gold standard for neuroscientific analyses of linear data (such as time series or evoked responses), an equivalent solution for circular data is currently not available. Therefore, in what follows, we describe an easy-to-use pipeline for continuous phase extraction and the joint analysis of respiratory and neural or behavioural data and introduce a novel extension of widely used cluster permutation testing for circular data.

### Methods synopsis

**Fig 1:**
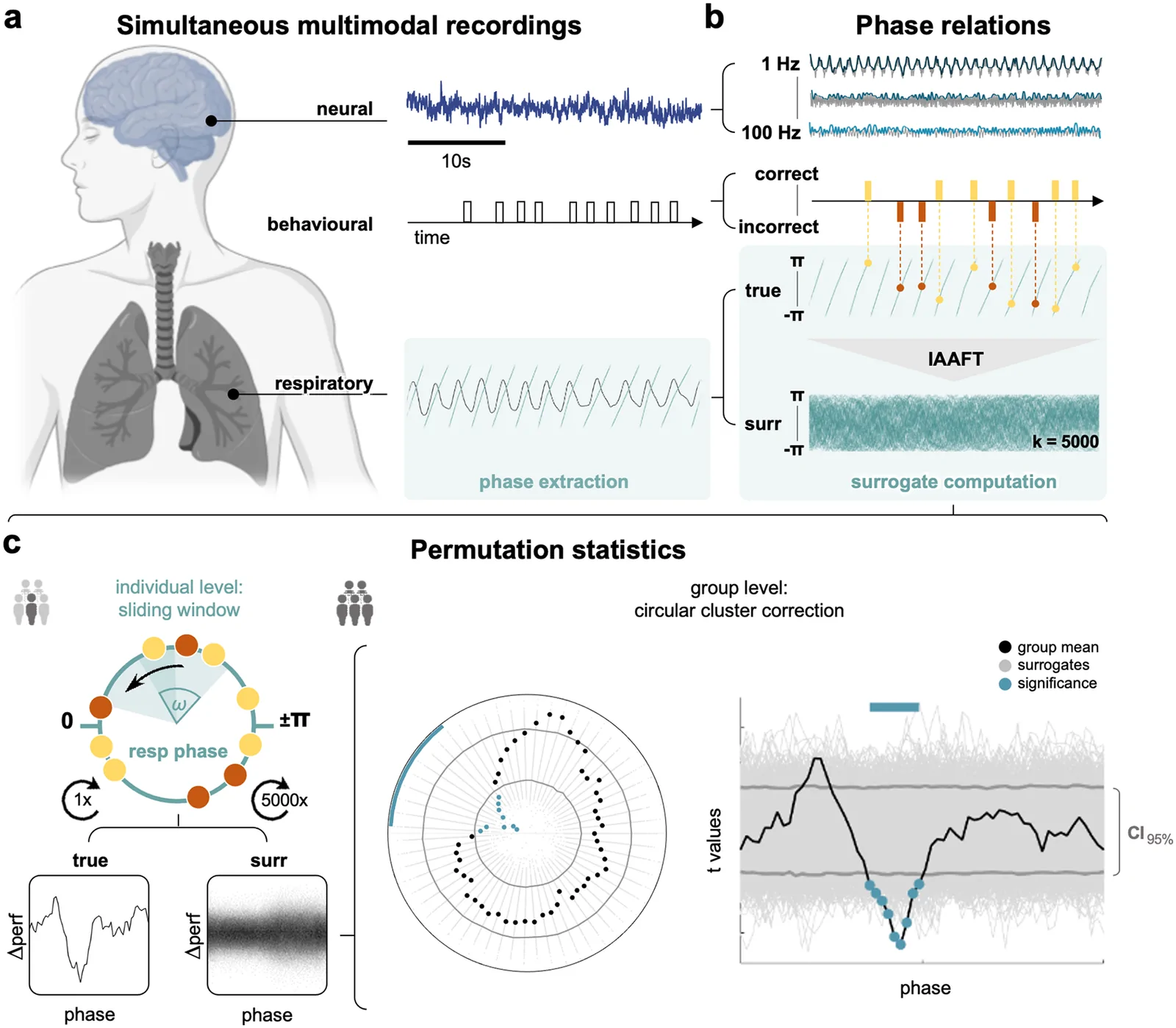
Example of a methods pipeline for behavioural/neural analyses based on quasi-continuous respiratory phase. **a,** Simultaneous recordings of neural and/or behavioural data and respiration allows for time-locked analyses of perception, cognition, and performance measures. Bottom panel shows extraction of continuous respiratory phase using two-point interpolation. **b,** Examples of domain-specific data processing. Top: Neural time series can be frequency-transformed for spectral analyses, resulting in time series of amplitude envelopes for frequencies within a predefined range (e.g., 1-100 Hz). Middle: Behavioural responses from any task can be categorized into correct vs incorrect responses. Due to high-precision triggers used in fMRI and M/EEG studies, each response can be assigned to the respiratory phase at which it was registered. Bottom: For statistical testing of respiration phase-locked changes in behaviour, a large number of surrogate phase vectors are computed using the IAAFT algorithm (see main text for details). **c,** On the individual level, trials are assigned to respiratory phase bins using a moving window approach. Performance metrics like hit rate are computed for each phase bin, both on the empirical phase vector and all surrogate phase vectors. On the group level, significant modulation of, e.g., hit rate ~ respiration phase is determined by circular cluster correction (see main text) whose results can be presented in the polar (top) or linear plane (bottom). Created in BioRender. Kluger, D. (2026) https://BioRender.com/m517rjb.

The main text of this tutorial follows a typical workflow going step by step from raw data inference statistics (Fig 1): Raw respiratory data are preprocessed to yield respiratory phase information. Based on this phase extraction, we then create a large number of surrogate respiratory phase vectors. Using the empirical and surrogate phase vectors, experimental outcomes of interest are assigned to (surrogate) respiratory phase bins, resulting in lower-dimensional matrices of outcome-by-phase bin. On the group level, these individual matrices are used for summary statistics and we finally apply circular cluster-based correction for multiple comparisons.

We start this tutorial with a brief overview of these steps and continue with more in-depth discussions and analyses of three key points, namely i) accurate extraction of continuous respiratory phase, ii) construction of a valid surrogate distribution for significance testing, and iii) multiple comparison correction for circular data. Throughout the tutorial, we highlight potential pitfalls and critical decision points and provide openly accessible software code to use, refine, and flexibly adjust our pipeline to a wide variety of research questions.

## Results

The basic premise of this tutorial are research questions related to systematic covariation between the respiratory rhythm and behavioural or neural outcome variables: *Does hit rate in a perception task fluctuate over the respiratory cycle? What is the relationship between alpha power and respiration phase?* To answer these questions, respiratory signals can be recorded time-locked to neural signals (e.g., from fMRI or M/EEG) and/or behavioural responses (e.g., button presses; Fig 1a). Respiratory modulation of neural activity can be quantified using phase-amplitude coupling [26]. Phase-amplitude coupling means that the phase, for example that of the respiratory rhythm, systematically covaries with the amplitude of neural activity, e.g., in the alpha frequency band. Whatever the final analysis might be, simultaneous recordings allow the analyst to extract continuous respiratory phase and either compute coupling metrics on continuous data or assign precise respiratory phase angles to single events like sensory stimuli or behavioural responses (Fig 1b). If we take the latter case as an example, events which occurred at similar points of the respiration cycle can then be grouped (or ‘binned’) together in order to quantify behavioural performance or the strength of neural responses within this subset of events, leading to respiration phase-resolved metrics of, e.g., hit rate. Particularly for a high number of phase bins, we refer to these signals as ‘quasi-continuous’, i.e., so densely sampled across the respiratory cycle that they approximate continuous phase (in contrast to a discrete inspiration vs expiration distinction). A very similar logic is applied in analyses of continuous time series (e.g., phase-amplitude coupling), which we will return to at a later point (see Fig 3).

Statistics on these phase-locked modulations are conducted by comparing empirical variation in hit rate across the respiratory cycle to variation in a null distribution. Since no canonical distribution can be used to determine whether the observed effects are larger than the effect found in a random partition of the data, one has to be constructed for the purpose of significance testing. Our approach is based on creating surrogate respiration time series for each participant using the so-called iterated amplitude-adjusted Fourier transform (IAAFT [27]). For each participant, a large number of IAAFT-transformed respiration time series can be computed. Similar to the empirical part of the analysis, surrogate respiration phase angles can be determined for time points of interest from the behavioural or neural time series (Fig 1b). This results in two data matrices: The first matrix contains one empirical phase angle for each event, the second matrix contains a large number (e.g., k = 5000) of surrogate phase angles for each event. Phase binning and analyses of interest (e.g., computation of hit rate within a certain number of phase bins) are then applied equally to the empirical matrix and the surrogate matrix (Fig 1c). In our example shown in Fig 1, this results in a 1 x 30 vector of empirical hit rate ~ respiration phase and a 5000 x 30 matrix of hit rate ~ surrogate respiration phase for each iteration of the IAAFT procedure per participant.

In keeping with inferential statistics conducted on non-circular data, bin-wise t-tests can be computed to compare the empirical outcome across participants to the distribution of surrogate outcomes. These results are then corrected for multiple comparisons since one t-test is conducted per phase bin. Critically, however, conventional approaches cannot account for the data’s underlying circularity, meaning they disregard the fact that phase bins covering angles close to ±π are in fact adjacent to one another. In Fig 1c, we illustrate the output of a novel approach we developed for cluster-based permutation testing of circular data. Put simply, this approach allows the analyst to assess statistical significance of phase-locked effects while controlling false positive rates in the special application of circular data. This step concludes our example analysis and yields a valid cluster-based statistic to answer the initial research question, e.g., whether or not hit rate significantly fluctuates across the respiratory cycle (see Fig 1c).

As previously mentioned, however, even the most simplistic analysis pipeline involves a plethora of methodological decisions between several alternatives being used in the field. Therefore, tracing the analytical steps outlined in this initial overview, the subsequent sections are focussed on the most critical points and offer detailed comparisons of widely used approaches for extracting respiratory phase and constructing surrogate distributions. We provide hands-on guidelines at these crossroads and finally present novel tools for circular cluster-based statistics which hopefully help move the field towards more robust, reliably, and replicable analyses of respiration-brain interactions.

To make our approach easily accessible, our tutorial paper is accompanied by software code (available for Matlab and Python) hosted on a publicly accessible GitHub repository (see section ‘Code availability’). Hence, while this paper lays out the practical framework of our proposed pipeline, we kindly refer the reader to the GitHub repository and its documentation for in-depth explanations of each function and practical example application scripts.

### Nexus 1: Accurate extraction of respiratory phase

**Fig 2:**
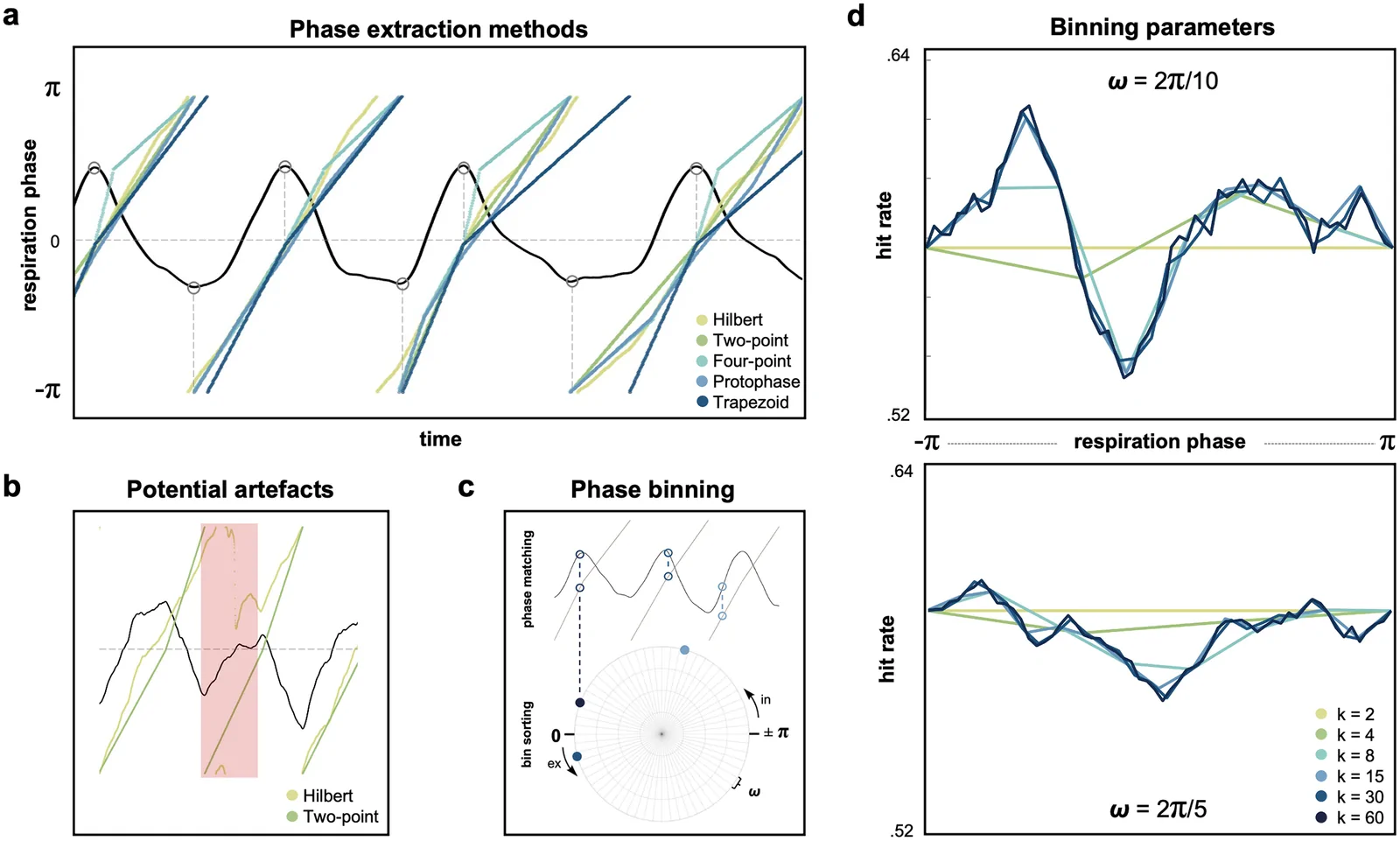
From raw respiratory recordings to quasi-continuous binned phase. **a,** Illustration of different methods for extracting continuous phase from the raw respiratory signal (black line). **b,** Particularly in noisier respiratory recordings, the Hilbert transform is prone to produce artefacts like phase jumps (red highlight). **c,** Following the extraction of continuous phase, phase information corresponding to events of interest (e.g., stimuli or responses) are assigned to respiratory phase bins for subsequent averaging within each bin. The more bins are defined to cover the respiratory cycle, the closer the analysis comes to using quasi-continuous phase information. **d,** The number and width of phase bins greatly influence the sensitivity of the analysis pipeline. Top panel shows phase-binned group-level hit rate for k = 2 to k = 60 bins with a fixed bin width (*ω*). Note that in this case, the sensitivity for detecting hit rate modulation more or less saturates at k = 15 bins. Comparing the top and bottom panels demonstrates how overall sensitivity decreases when bin width *ω* is doubled.

Available approaches for extracting respiratory phase (Fig 2a) include Hilbert-based methods [28], interpolation algorithms [9], and protophase extensions [29], of which the Hilbert transform is by far the most widely used. Two-point interpolation is based on identified peaks and troughs in the respiratory signal and linearly interpolates respiratory phase between these landmark points. Depending on how respiration was recorded, peaks and troughs can resemble maxima and minima of thoracic circumference (recorded with a respiratory belt), respectively, or the points of strongest and weakest air flow (in flow meter data). In our respiratory belt examples, the absolute minimum identifies peak expiration, i.e., the lowest amount of air in the lungs before the next inspiratory phase. The recording method has no implications at all for subsequent steps in our pipeline other than the phase angles assigned to peaks and troughs before interpolation (if used). Four-point interpolation additionally considers inflection points in the time series, whereas protophase approaches improve the Hilbert transform in an iterative approach. Depending on the levels of noise and/or smoothing in the respiratory time series, each of these approaches will yield slightly different phase vectors. While these differences may intuitively appear miniscule, potential artefacts like restricted range or phase jumps sometimes observed for the Hilbert transform (Fig 2b) will influence the validity of phase-related analyses. This is of particular importance as the use of phase information ought to go beyond a simple discretisation into inspiration vs expiration: Since the extracted respiratory phase vector will subsequently be divided into a certain number of phase bins (Fig 2c), artefacts or inconsistencies in the vector may cause events or time points being assigned to the wrong phase bin. The more fine-grained the bins are set up (i.e., the smaller the bin width *ω*), the more likely this assignment error becomes.

Of note, interpolation approaches require a reliable detection of peaks and troughs in the raw respiratory trace. This detection can at times be ambiguous, e.g., in the case of transient breath holds (i.e., plateaus in the respiratory data). Here, we recommend assigning the peak/trough to the absolute maximum/minimum or the centre of the time frame for which the plateau is observed. Furthermore, since the absolute range and variability of raw respiratory data will differ greatly between participants, normalisation of individual respiratory time series helps mitigate interindividual variance. Among other things, this allows for the exclusion of artefact-laden breathing cycles (e.g., sighs with extreme amplitudes), and refining peak detection algorithms with sensible thresholds so that neither too few nor too many peaks are marked as such. Regardless of the phase extraction method used, slight smoothing with a kernel of around 10% of the average cycle duration (i.e., around 500 ms) will reduce high-frequency noise in the respiratory time series without negatively affecting the detection of peaks and troughs (for examples, see [16,30]).

Once continuous respiratory phase has been extracted, the analyst has to decide on the granularity of their subsequent analysis. This is when, too often, the breathing cycle is discretized into inspiration vs expiration despite both numerous prior studies [4,9,16,19,22–24,31–33] and systematic parameter comparisons (Fig 2d and 2e) demonstrating that nuanced, phase-specific effects are common but cannot be picked up with a binary comparison. Instead, respiratory phase angles at time points of interest, e.g., the presentation of a stimulus or a behavioural response given by the participant, should be assigned to an arbitrary number of phase bins - the more phase bins are used, the more closely the final analysis approaches quasi-continuity.

For the phase binning approach, the analyst defines i) the width of the moving window (ω) as well as the number of phase bins used (k; see Fig 2c). Together, these parameters determine how fine the resolution of the outcome variable across the respiratory cycle ought to be. As a practical example, imagine that hit rates ought to be computed for k = 30 phase bins across the cycle with a moving window width of ω = 2π/10 = 36°. In an iterative approach, hit rate is first computed across all trials presented at respiratory phases ranging from 0° ± ω/2, i.e., -18° to 18°, and assigned to the bin centre at 0°. The analyst then moves forward in steps of Δφ = 360°/k = 360°/30 = 12°, applies the same window width of ω = 36° (this time reaching from 12° ± 18°, i.e., from -6° to 30°), computes the hit rate within this subset of trials, and assigns this value to the bin centre at 12°. This procedure is repeated k = 30 times until the entire respiratory cycle has been covered (see Fig 1c).

During this procedure, the overlap between neighbouring phase bins results from the relationship between the number of bins (k) and the width of the moving window (ω): Since the moving window is defined symmetrically around the center of each phase bin, it follows for each combination of k and ω that whenever Δφ < ω, the incremental steps of the moving window are smaller than its width, which results in overlap. For Δφ = ω, neighbouring phase bins would be exactly aligned, while Δφ > ω will likely lead to the adverse scenario that some trials are not considered in the analysis at all (since the moving window will not cover all phase angles).

The definition of key parameters (e.g., k, ω) depends on both the research question and the available data: How are trials distributed across the breathing cycle? Does the number of trials suffice for fine-grained binning? Are certain phases of particular interest, e.g., transitions between inspiration and expiration or time points of strongest air flow? Ideally, to answer these questions, analysts should use independent data to systematically vary parameters like the number and width of phase bins in order to identify those which yield the highest statistical power. This combination of parameters can then be applied in the analysis of the main dataset. As a general guideline, we suggest parameter selection in either pilot data or an independent subset with a sample size of around 10–20% of the desired final sample. Fig 2d shows an exemplary comparison of critical settings within a subset of N = 18 participants from a behavioural experiment: While it is difficult to provide definitive rules for a minimum number of events per phase bins, experimenters are generally well-advised to treat phase bins as factor levels and ensure that each bin contains a number of events similar to event-related analyses in M/EEG research, i.e., no fewer than 50 events [34]. At any constant bin width *ω,* sensitivity for finding respiration phase-dependent modulation of an outcome variable (e.g., hit rate) increases with the number of overlapping phase bins. At some point, however, further increasing the number of bins only adds noise and computations within each bin are more prone to be influenced by outliers due to a small number of trials. On the other hand, and as expected, increasing the width *ω* lowers the overall sensitivity for phase-specific effects. Hence, both binning parameters should be optimized either in testing data or on the grounds of prior hypotheses before computing phase-locked effects on empirical and surrogate data (e.g., based on IAAFT). Since great care has to be taken in constructing an adequate surrogate distribution, the following section validates our IAAFT pipeline against other commonly used surrogate approaches to raise awareness regarding their potential limitations.

### Nexus 2: Adequate surrogate distributions using IAAFT

Any valid null distribution for phase-based analyses requires that the surrogate phase time series be statistically independent of the original phase, disrupting any systematic phase-outcome relationship. At the same time, it is essential to preserve some defining characteristics of the original time series, including its mean, variance, spectral composition, and temporal autocorrelation [35]. Only if both requirements are met can differences between empirical and surrogate results be meaningfully interpreted.

Unfortunately, widely used approaches like circularly shifting or shuffling segments of the original time series fail to fulfil these requirements and involve somewhat arbitrary parameter decisions (see below). While circular shifting keeps the autocorrelation of the signal intact, it often does not generate a surrogate time series whose phase is truly independent of the original time series. This is due to the limited number of possible different surrogates under the respective constraints so that the surrogate phase vectors themselves might be highly correlated. Whenever that happens, the analyst is likely to underestimate any empirical phase-dependent modulation since the surrogate distribution against which they are tested too closely resembles the true data. Of note, deciding on central parameters such as the extent of circular shifting is not straightforward, especially for a highly regular signal like respiration or (even more so) gastric rhythms.

**Fig 3:**
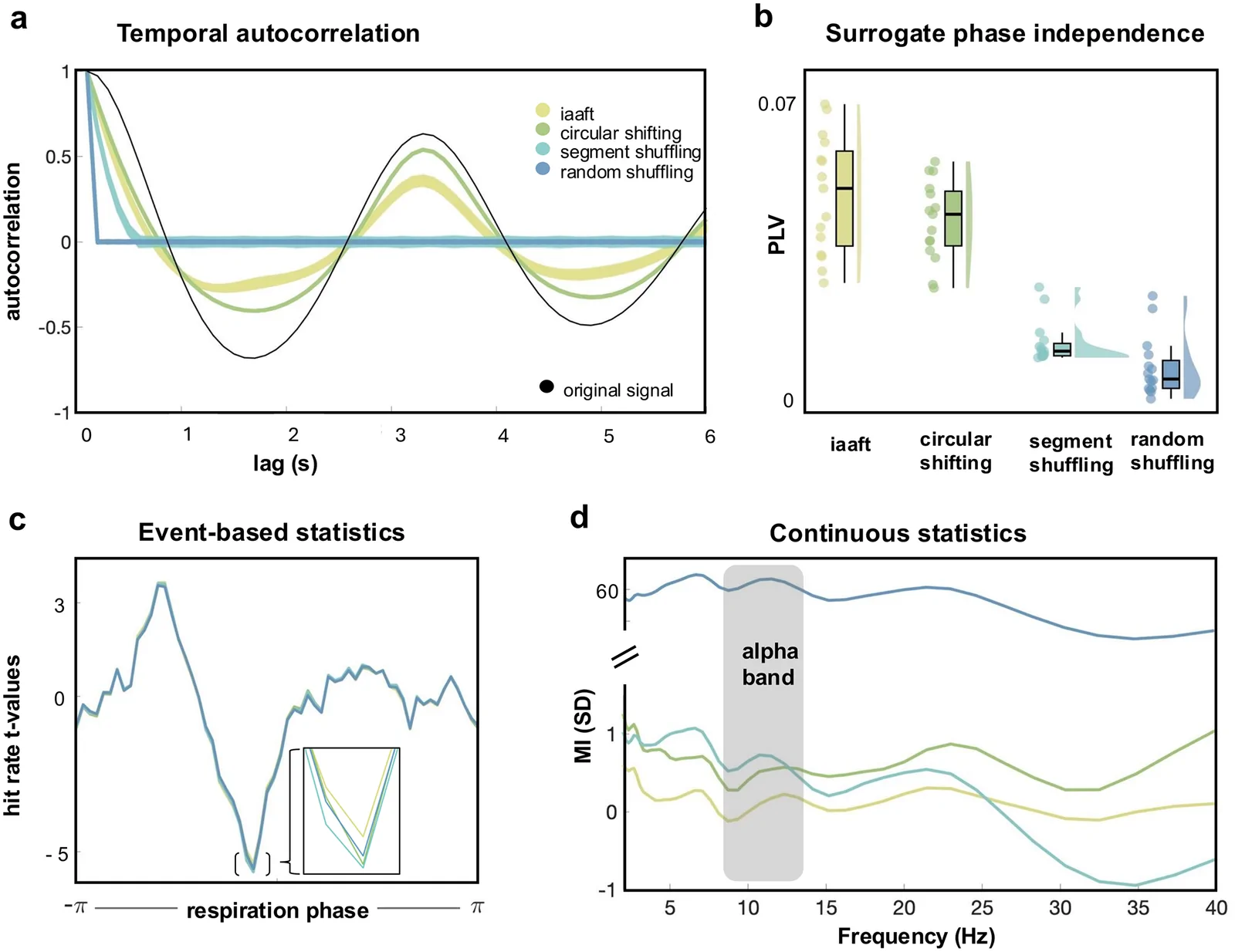
Implications of different surrogate procedures. **a,** Comparison of temporal autocorrelation functions for the original data and surrogate phase vectors yielded by IAAFT, circular shifting (minimum shifts of 30s), as well as shuffling of segments (length of 500ms) and single samples, respectively. Note that only the IAAFT and circular shifting retain the autocorrelation of the original data. **b,** Group-level (N = 18) phase independence between original and surrogate data for all four methods of surrogate construction, computed as the phase-locking value (PLV). Note that the shuffling of single samples or short data segments leads to highest independence (i.e., lowest PLV) but destroys the temporal autocorrelation function (see panel **a)**. In contrast, IAAFT and circular shifting result in high independence as well (i.e., very low PLV overall), but do retain the original autocorrelation. **c,** Group-level (N = 18) distributions of hit rate ~ respiration phase (shown in t-values) for all four methods of surrogate distribution. Note that there is no discernible effect of surrogate method on the group-level results (but see main text). **d,** In contrast, analyses of continuous data (such as MI, expressed here in units of standard deviation of surrogates) suffer from false positives introduced by overly liberal surrogates constructed from random shuffling of single events. Alpha frequency band (8-13 Hz) marked in grey.

Random shuffling of time series segments suffers from a similar limitation, namely deciding on the parameter defining the length of the to-be-shuffled segments. While the shuffling approach will perform better in regard to creating independent phase distributions (compared to circular shifting), it will not sufficiently retain temporal dependencies of the original time series. These changes in temporal autocorrelation introduced by random shuffling will cause the analyst to overestimate their phase-locked effects and ultimately render this approach unsuitable for the generation of appropriate surrogate data [36]. As we illustrate below, this limitation affects analyses of continuous time series (e.g., phase-amplitude coupling) more strongly than event-based analyses (see Fig 3c and 3d).

We propose the creation of a surrogate distribution using IAAFT on the original respiratory time series. In short, the algorithm creates randomized copies of an original time series which are constrained to have the same power spectrum (and thus the same linear autocorrelation) and the same amplitude distribution (histogram) as the original data. After an initial guess for surrogate time series, the IAAFT sequentially applies both the spectral and the amplitude constraint and keeps the newly generated surrogate time series only if both constraints are met.

In the exemplary data processing and surrogate generation script provided with the code repository for this tutorial, the IAAFT surrogate generation is adapted using the *generate_surrogate_iaaft* function provided by the NSE Laboratory of the Physics Department at the University of Trento ([37]; see https://github.com/LeonardoRicci/iaaft). Again using the z-scored respiration time series as its input, it generates a single surrogate time series.

The fact that the IAAFT is applied to the original respiratory time series has two major implications: First, it underscores the importance of extracting respiratory phase with a valid approach (i.e., preferably not a simple Hilbert transform) since phase extraction has to be reliably performed many times per participant. Second, it simplifies the pipeline to some extent since any manual adjustments such as threshold definition for peak detection remain valid for all the surrogate iterations because the amplitude range remains unchanged from the original time series (see the second IAAFT constraint above).

Different criteria can be applied to the resulting surrogates in order to evaluate the performance of the IAAFT approach. Recall that the IAAFT’s main practical advantage is the absence of arbitrary parameter settings, particularly compared to circular shifting which is the other approach designed to retain the empirical temporal autocorrelation. Hence, the first evaluation criterion for any of our surrogate approaches (IAAFT, circular shifting, segment shuffling, random shuffling) is whether or not their respective outputs are true to the autocorrelation of the empirical data. As can be seen in Fig 3a, only circular shifting and IAAFT retain the autocorrelation characteristics of the original time series whereas neither shuffling approach does (data taken from a randomly selected participant). Naturally, and as desired, this reliably destroys any phase relationship between the original and surrogate time series for both segment shuffling and single sample shuffling, but IAAFT and circular shifting yield very good phase independence as well (Fig 3b): Here, we computed the mean phase-locking value (PLV) between empirical and surrogate data in a group of N = 18 participants. This metric ranges between PLV = 0 (no phase relationship) and PLV = 1 (perfect phase correlation), and we observed very low group-level means around PLV = 0.05 for IAAFT and circular shifting, demonstrating that both approaches provide phase-independent surrogate distributions while controlling for temporal autocorrelation.

It is important to note that the extent to which the choice of surrogate methods will influence results critically depends on the type of research question. Coming back to our initial examples, the question regarding hit rate changes over respiration phase entails an *event-related* analysis: Over the duration of the experiment, single trials are presented (ideally) uniformly distributed across the respiration cycle, so that each trial can be assigned its corresponding respiratory phase (e.g., at the time point of target or response onset). If (and only if) trials are uniformly distributed so that there is no systematic relationship between trial onsets and respiration phase, creating a surrogate distribution by shuffling phase across trials is perfectly valid and the final results of hit rate ~ respiration phase are almost identical across the four surrogate methods we discuss here (Fig 3c). This is due to the fact that the temporal autocorrelation of the respiratory signal becomes less important the more uniformly events are sampled across the respiratory cycle. Therefore, any study interested in respiration-related effects ought to conduct pilot measurements to exclude any bias introduced by the timing of the experimental protocol itself, e.g., trials being presented more frequently in one respiratory phase than another. In case a non-uniform distribution of trials across the respiratory cycle is noticed a posteriori, shuffling phase across trials does no longer provide a valid surrogate distribution and an alternative approach (e.g., IAAFT) has to be implemented.

In stark contrast to event-based analyses, the choice of surrogates becomes much more important for research questions which make use of continuous respiratory data. Analyses of phase-amplitude coupling, for example, quantify the extent to which the amplitude of neural activity (e.g., in the alpha band) is coupled to respiratory phase. Metrics such as the modulation index are computed with a moving window approach along the continuous respiratory phase vector. Since the null effect of any empirical modulation is then determined by conducting the same moving window analysis on continuous surrogate phase vectors, it is vitally important that this surrogate distribution is validly constructed: Ideally, each surrogate phase vectors destroys the relationship between time points and phase in the original time series but keeps central statistical properties intact. To reiterate, shuffling single samples destroys the empirical temporal autocorrelation and therefore leads to a vast overestimation of phase-amplitude coupling across the entire frequency band (Fig 3d). In this case, the answer to our initial research question would merely be based on false positive results due to invalid statistical testing.

Hence, analyses of continuous respiratory signals are very instructive for illustrating the influence of surrogate methods choices: Although segment shuffling yielded only slightly more liberal MI spectra than circular shifting and IAAFT in our example (see Fig 3d), we deliberately chose relatively long segments of 500ms. In addition to not retaining the temporal autocorrelation, validity of segment shuffling critically depends on the arbitrary choice of segment length and will approximate the false positive tendencies of random shuffling at shorter segment definitions. Circular shifting yields comparable (albeit slightly more liberal) results in our MI analysis example because it retains the original temporal autocorrelation; however, this method comes with the caveat of having to define the free parameter of shifting distance. Shorter distances mean greater similarity of surrogate and original data, which leads to conservative estimates. With highly regular signals (such as respiration), it is difficult to define meaningful constraints on the range of acceptable shifting distances. This is why we suggest and have previously validated [12,14] the IAAFT algorithm as a parameter-free method for the creation of surrogate data.

In the final step of the analysis, we employ a newly developed circular cluster correction approach to determine statistical significance of differences in our outcome variable across empirical and surrogate respiration phase, respectively. This is a critical contribution to our analysis pipeline: Even in the ideal case that, as we have shown above, an analysis of events uniformly distributed across the respiratory cycle is not influenced by the surrogate method (Fig 3c), there is still no reliable approach available for significance testing across a circular independent variable.

### Nexus 3: A novel approach for circular cluster-based permutation testing

Conventional cluster-based permutation tests assuming a non-circular independent variable fail to account for the wrap-around continuity inherent to circular data. To overcome this failure, we propose a new clustering procedure based on complex representations in the polar plane that takes the circular nature of phase data into account and reliably controls error rates in phase-related analyses. In this tutorial, we use examples of significance testing hit rates across respiratory phase bins. This approach, however, can be used to examine any variable of interest fluctuating across a circular predictor. While highly instructive papers have previously outlined the theoretical, formal, and practical aspects of cluster-based permutation testing [21,25,38,39], we want to briefly describe our proposed extension for the special use-case of circular data.

At this stage, we assume that respiratory phase has been extracted from the raw recordings (e.g., with two-point interpolation, Nexus 1), a surrogate distribution has been constructed using the IAAFT procedure (Nexus 2), and the outcome variable has been binned according to both empirical and surrogate phases. As a result, we have our outcome variable across one single vector of empirical and a large number (e.g., k = 5000) of surrogate phase vectors. Now, in a first step, both surrogate and empirical data are *z*-scored within each phase bin, based on the distribution across all surrogate and empirical values. Subsequently, we calculate *t*-values for each bin using the combined surrogate and empirical value distribution by dividing the mean across subjects by the across-subject standard deviation and scaling the result by the square root of the number of subjects.

Specifically, we apply


$$\begin{array}{c} t\;=\frac{\sqrt{N\;}\;\underset{―}{x}}{sd} \end{array}$$ (1)

to the concatenated matrix of *z*-scored surrogate and empirical values, where N is the number of subjects, $\underset{―}{x}$ is the mean across subjects, and sd is the standard deviation across subjects. Using the empirical cumulative distribution function on the combined distribution of surrogate and empirical *t*-values, the lower bounds for cluster definition are computed for each bin as the value corresponding to a cumulative probability of ≤ 2.5%. The upper bound for each bin is defined as the value corresponding to a cumulative probabili*t*y of ≥ 97.5%, respectively.

Second, both empirical values and the values for each surrogate iteration *j* are converted to a complex representation in the polar plane:


$$\begin{array}{c} c_{j}\;=\;m_{j\;}*\;e^{i\;\theta_{j}}\;=\;m_{j}\;*(\;cos(\theta_{j})\;+\;i*sin(\theta_{j})) \end{array}$$ (2)

with c being the vector of complex representations, m being the vector length in the polar plane, set to 1 if the data point exceeds the cluster thresholds and 0 otherwise, and θ denoting the phase angle. Clusters are then defined as sets of adjacent points with the minimal non-zero angular distance, and the cluster statistic for each cluster is computed as the sum of all points in this cluster. Recall that this procedure is executed for empirical and surrogate values, resulting in clusters for a single empirical distribution and a large number of surrogate distributions of outcome ~ phase bins.

Finally, permutation *p*-values for each empirical cluster are calculated as the proportion of surrogate cluster statistics with an absolute value greater than or equal to the observed cluster statistic. As a practical implication, this is where the number of surrogates used (see Fig 1b) determines the range of possible *p*-values, i.e., a cluster-based permutation with only *k* = 500 surrogates cannot yield *p* < .002. Note that for group-level cluster inference, additional correction methods such as finite-samples correction ([40]) can be implemented, if desired.

### Simulation-based validation of the circular cluster-permutation procedure

To validate our proposed novel method of circular-clustering, we conducted simulation analyses to assess the probability of both Type I and II errors (false positive and false negative, respectively) of the approach. Modelling the probability of a correct response to a stimulus as a function of respiratory phase, we systematically varied effect magnitude, variability in response probability across trials, and effect phase coherence across subjects (see Supplemental Material for details on model definition and parameter space). In addition to t-values as a measure of relative dispersion, we included a more robust measure based on the interquartile range and benchmarked our clustering approach against the Omnibus correction for family-wise error rates [41] (but see [42] for limitations). This resulted in four possible combinations of dispersion statistics and multiple comparison correction methods, with each combination and parameter being tested 20 times for consistency.

Additionally, to provide an overview of the statistical power of our approach, we also computed the probability of reporting an existing effect over a series of experimental and preprocessing choices.

### Benchmarking

**Fig 4:**
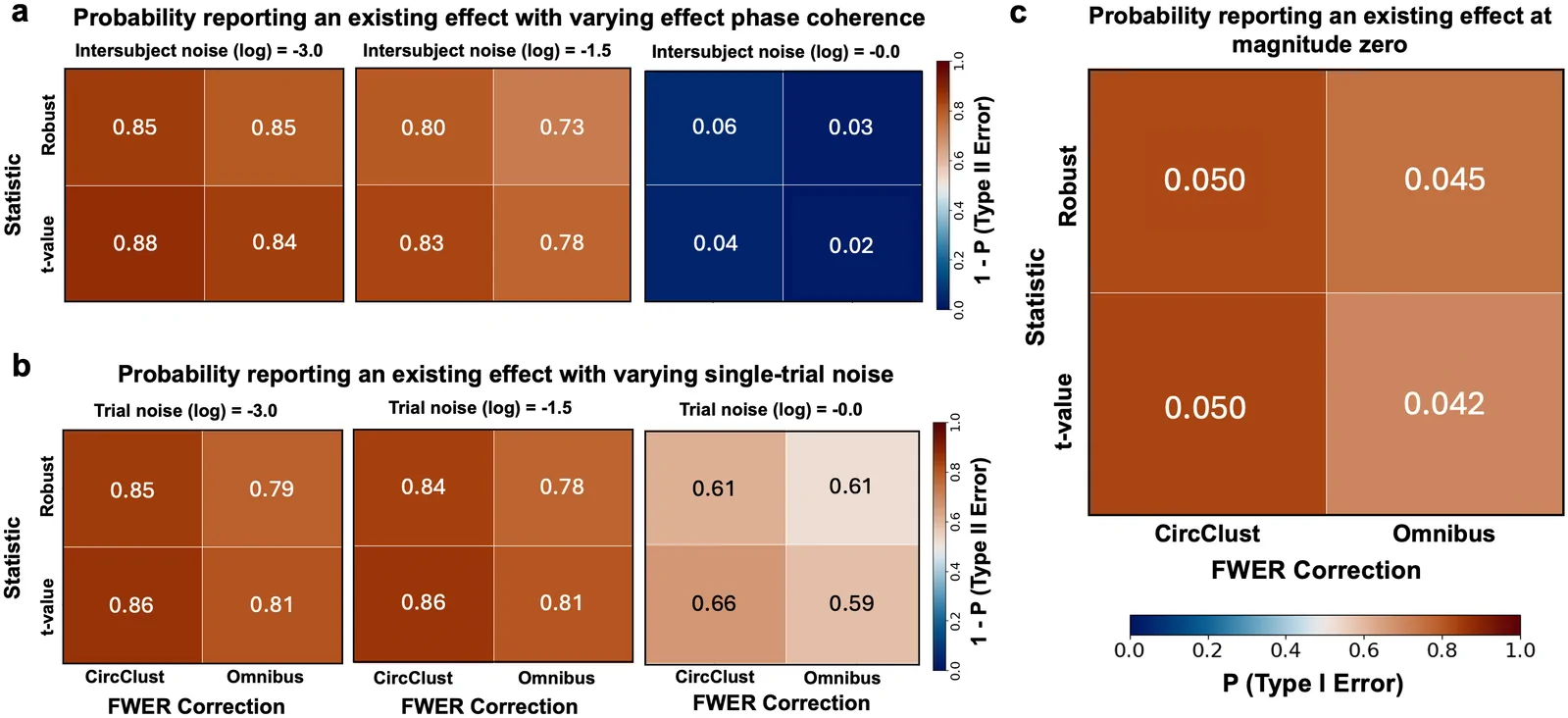
Statistical performance across Type I and Type II errors. **a**, Probability of reporting an existing effect (Type II Error) as a function of the Family Wise Error Correction (FWER) method (circular clustering vs Omnibus), the statistic adopted (t-value or Robust), and the intersubject noise level simulated (natural log). **b**, Probability of reporting an existing effect as a function of the FWER method, the statistic adopted, and the trial noise level simulated (natural log).

Overall, when comparing varying levels of noise in phase coherence of the true effect across subjects, the combination of circular clustering and t-values yields the lowest probability of Type II error at low to intermediate noise levels (Fig 4a, for the Type II error rates at all simulated intersubject noise levels refer to S1a Fig). At high intersubject noise level, i.e., when the phase coherence of the effect is strongly disrupted across participants, the capacity of detecting a true effect drops for all the analytical approaches.

Next, we systematically varied single-trial noise levels, i.e., the variability in response probability across trials within each subject. To eliminate a uniform confound that would lead to a misleading low probability of reporting a true effect, we excluded the two highest levels of intersubject noise from the simulation parameters. As shown in Fig 4b, our approach combining t-values and circular clustering was again most likely to detect an existing effect for all noise levels (for the Type II error rates at all simulated trial noise levels refer to S1b Fig).

Furthermore, we evaluated the Type I error across the different analytical approaches by computing the probability of reporting a significant result when the simulated effect magnitude was zero. Consistent with the previous results indicating that the Omnibus correction is the more conservative approach, this correction yielded a lower probability of Type I Error than our circular clustering correction. Crucially, however, the circular clustering correction still keeps the Type I Error at the expected level of ~ 0.05 (see Fig 4c).

### Statistical power across experimental and preprocessing choices

In addition to the simulation-based benchmark, we assessed the statistical power of our circular cluster permutation test across a range of experimental and preprocessing choices, some of which we have discussed in detail above. Specifically, we evaluated the probability of reporting an existing effect at varying magnitudes, over different sample sizes, numbers of trials, bins, and moving window widths.

**Fig 5:**
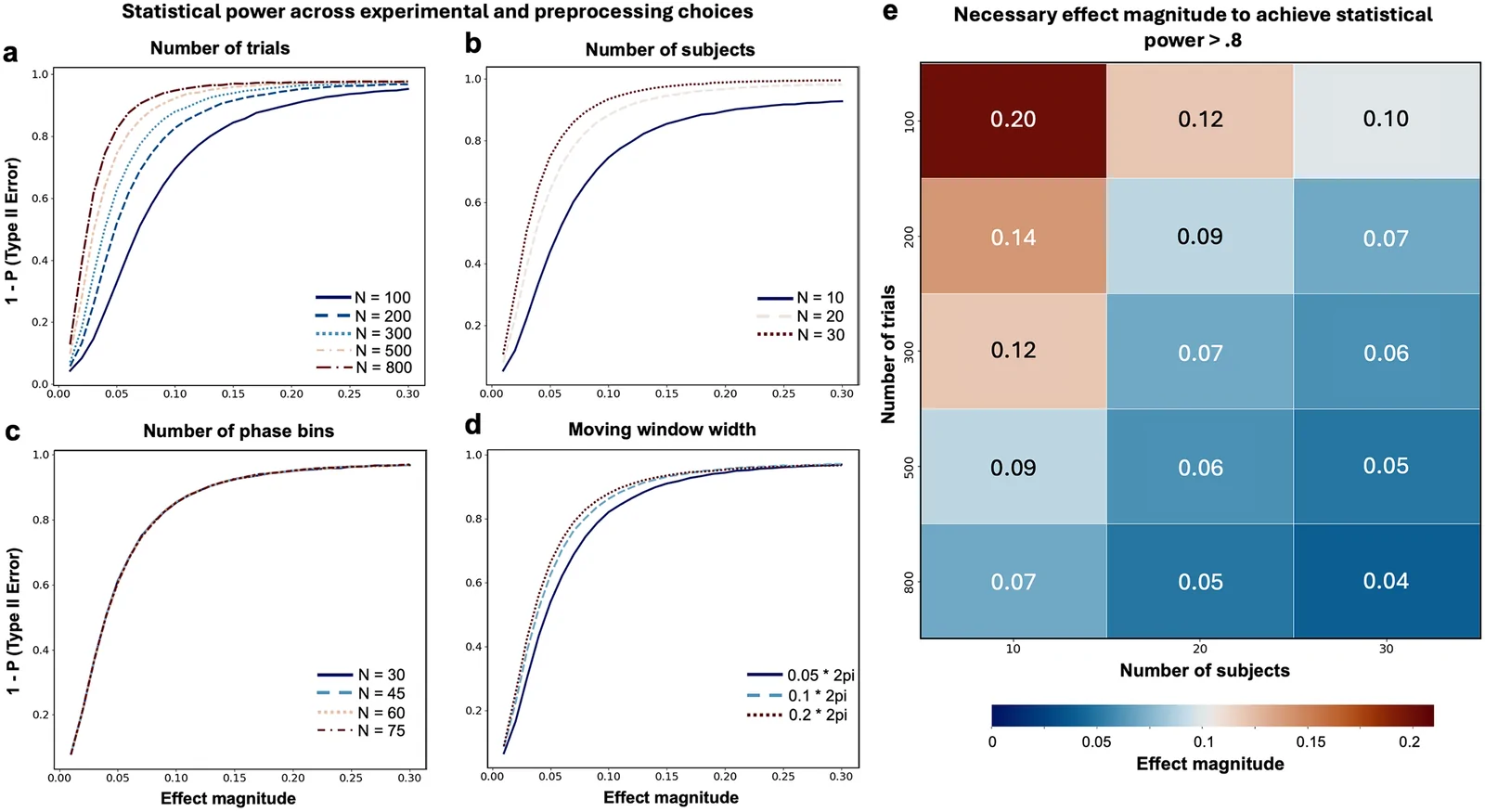
Statistical power across experimental and preprocessing choices. **a**, Probability of reporting an existing effect (Type II Error) as a function of effect magnitude and trial number. **b**, Probability of reporting an existing effect as a function of effect magnitude and number of subjects. **c**, Probability of reporting an existing effect as a function of effect magnitude and number of phase bins. **d**, Probability of reporting an existing effect as a function of effect magnitude and phase bin width. **e**, Minimal estimated effect magnitude necessary to achieve a statistical power above.8, as a function of the number of trials and of subjects.

As shown in Fig 5a and 5b, respectively, the number of trials per subject as well as the sample size have a critical impact on the probability of detecting a true effect. For both parameters, power increased with effect magnitude; however, this increase was much more gradual with lower trial numbers and smaller sample size. In line with the considerations on phase binning in Nexus 1, increasing the number of phase bins beyond k = 30 does not impact the probability of reporting an existing effect across different effect magnitudes (see Fig 5c). Remarkably, a wider moving window leads to an increased probability of a true positive result (see Fig 5d). This effect may be expected in a quasi-ideal scenario where the effect of interest is a noise-corrupted sinusoid: A wider moving window leads to increased smoothing of the underlying signal, improving the signal-to-noise ratio. As discussed in Nexus 1, the benefit of smoothing should be weighed against the possibility that wider bins may obscure relevant features of the underlying data.

Lastly, Fig 5e provides a closer overview of the tradeoff between the number of trials and subjects to facilitate estimates of expected statistical power given different effect magnitude values. We estimated the minimal simulated effect magnitude necessary to achieve a statistical power exceeding 0.8. This threshold ranged from 0.20 for 10 subjects completing 100 trials each to 0.04 for 30 subjects completing 800 trials each, corresponding to hit-rate modulations of approximately ±0.20 and ±0.04 around the mean, respectively. The parameter space of this simulation was rather broad, thus we provide all simulation code in the accompanying GitHub repository and invite the reader to rerun the simulation with parameters and models as closely adherent to their specific case as possible.

## Discussion

In this tutorial paper, we present a unified methodological approach for phase-coupling analyses tailored to (but not limited to) applications in the field of respiration-brain interactions. We propose an easy-to-use, generalisable pipeline that can flexibly be implemented for any phase-locked analysis of neural, behavioural, or peripheral data. Our approach advances the status quo of brain-body research in two central aspects: First, we implement a unified way of generating surrogate phase distributions for statistical analysis. Second, we present a novel extension of cluster permutation procedures specifically for circular data in order to control error rates in phase-related analyses. In the tradition of ‘Ten simple rules’ papers, we close with synoptic recommendations which we hope will lead towards greater reliability, overall robustness, and reproducibility in brain-body neuroscience.

1. *Do not compromise research questions for the sake of simpler statistics.*

Mechanisms underlying the interactions of respiration and brain activity or behaviour are likely much more nuanced than a mere distinction between inspiration and expiration (see rule #7). Ask the question you are truly interested in and adjust your analysis pipeline accordingly, not vice versa.

2. *Be mindful of domain-specific caveats that come with analyzing cross-frequency brain-body data.*

While physiological rhythms are generally slower than brain rhythms, each organ system (heart, lungs, stomach) has its specific caveats, so do not expect, e.g., a respiratory pipeline to work on gastric data just because both signals are circular. This is a critical point, so we go into a little more detail on distinctions and commonalities below.

3. *Physiological signals vary greatly between participants - plug and play solutions will likely not work for all data.*

We suggest not to rely on a simple Hilbert transform for phase extraction, and interpolation approaches will require a little manual optimisation regarding peak detection. Running peak detection once with fixed parameters will work for most participants, and you can adjust parameters as required after checking the outputs (see rule #6). Note that normalisation is a great way to speed up that process (see rule #4).

4. *Normalisation is a convenient way to deal with interindividual variability and help setting the right parameters, e.g., for peak detection.*

Participants breathe at different rates and volumes and your data quality further depends on the device you used, how it was positioned, and much more. Normalising (z-scoring) the raw respiratory data goes a long way in identifying artefact-laden breathing cycles and defining peaks and troughs for interpolation.

5. *Compare different methods for phase extraction and decide which one fits best in your analysis.*

Although we are suggesting the use of interpolation and have had good experience with it, there may be research questions for which an equal distribution of phase between inspiration and expiration would be advantageous. Again, the methods follow the question.

6. *Visualize outputs from your preprocessing pipeline at every step of the way.*

This is critical for making decisions on preprocessing parameters and later steps in the analysis. Make sure you have high quality respiratory data, check the output of your peak detection, and run sanity checks on phase vectors.

7. *Collapsing continuous respiratory phase into an inspiration vs expiration dichotomy will almost always be the less sensitive (and sensible) choice.*

Not only is the mechanism you are interested in probably much more complex than that (see rule #1), but you are almost certainly missing nuanced modulation effects which can only be observed at specific points of the respiratory cycle.

8. *Parameters for phase binning should ideally be fixed in a separate dataset.*

The number, width, and overlap of your phase binning will greatly influence the sensitivity of your analysis. If no training dataset is available, decide on the number of bins by constraining the lowest number of trials you want per phase bin.

9. *Shuffling single time points is not a valid permutation approach for continuous analyses and will yield a large number of false positives.*

This is particularly true when your events are not uniformly distributed across the respiration cycle or when your research question calls for the analysis of continuous respiratory data (e.g., phase-amplitude coupling). Using the IAAFT-based approach yields valid surrogate data, irrespective of any particular use case.

10. *Make sure to account for the circular nature of phase data and avoid tests or corrections which assume linear data.*

Conventional inferential statistics do not account for the circularity of your phase data. The circular cluster-based permutation approach we propose here provides a tool for significance testing and multiple comparison correction that can be used in any analysis of circular data distributions.

Given the demonstrably strong influence of methodological choices outlined in this paper, we further suggest to clearly report key parameter choices in studies of neural or behavioural data as a function of physiological signalling. At minimum, these should include the method of phase extraction (with exemplary visualisation), phase binning (with detailed information regarding bin number and width), surrogate generation, and correction for multiple comparisons. In the interest of reproducible science, these choices can be made a priori in a preregistered analysis protocol and/or the analyst provides sensitivity analyses across a range of sensible parameter values.

We want to emphasize that some aspects of our proposed pipeline generalize well to new applications whereas others are relatively specific to respiration-brain coupling. As outlined above, the cluster-based permutation approach extends well beyond any particular research question and as such is not limited to respiration phase-related analyses. Rather, it provides a general statistical framework for the analysis of behavioural or neural data in relation to any circular signal (e.g., recorded across respiratory phase, visual angles, or time of day) based on established practices in the field of neuroscience. As we note at the outset, however, the properties of physiological signals recorded from the heart, lungs, and stomach, are very diverse with regard to their respective frequencies. As a consequence, the systematic comparison of phase extraction methods was conducted specifically for respiratory analyses and will not hold for cardiac data (which are discretised into diastole and systole) or gastric data (which can usually be handled with a simple Hilbert transform). Moreover, the proposed IAAFT-based approach will likely not be beneficial for gastric recordings, for example: The low frequency combined with high regularity of the signal means that the IAAFT algorithm will have a hard time generating adequate surrogates whose phase is not highly correlated with that of the original signal. In practice, this means that after preprocessing of the gastric signal, another approach should be chosen to generate surrogate data for subsequent statistical comparisons. If the analyst then wants to quantify whether behavioural, neural, or other physiological outcomes show meaningful variability across the gastric cycle, they can easily integrate their externally generated surrogate vectors in our provided code for cluster-based permutation testing.

In whichever use case of cluster-based statistics, caution should be exercised regarding the interpretation of the precise phase angle for which significant effects are observed. Naturally, cluster-based permutation statistics on circular data involve the same caveats as their non-circular counterpart: Cluster-based permutation tests control the false alarm rate only under the so-called ‘omnibus null hypothesis’, meaning that any modulation over time, space, or (in the current use-case) phase is tested against the null hypothesis that there is *no meaningful difference anywhere* in the data. Consequently, it explicitly does not provide statistical evidence for an effect at certain latencies (in linear data) or phase angles (in circular data). For the interpretation of cluster-based inference statistics, this means that, e.g., significant *p*-values for a cluster ranging from -90° to -30° should not be reported as ‘significant modulation between -90° and -30°’, simply because the procedure does not make any statistical claim about the cluster extent. Rather, the analyst should report this finding in a manner that is less phase-specific, for instance: ‘Non-parametric cluster-based permutation testing indicated that there is a group-level effect of respiratory phase on hit rate (*p* <.05) with unknown sign, corresponding to a cluster found during the inspiratory phase’. For excellent discussions of this limitation, see [21,38,43]. Note that confidence intervals for onsets and offsets of effects can be gained from a hierarchical sampling approach [44].

For now, the purpose of this tutorial was to provide an overview of a usable analytical pipeline particularly for inexperienced analysts in the rapidly growing field of brain-body neuroscience. As for intended future applications of our circular cluster-based approach, it is our hope that both ourselves and the community will develop the algorithm further and implement new functionalities as needed. This includes features such as higher-dimensional correction, e.g., in analyses of alpha power ~ respiration phase conducted across a set of M/EEG channels. In this case, the number of multiple comparisons increases further and cluster-based statistics could (but do not have to) be computed across both phase and space simultaneously. Note that in such an application, the aforementioned limitations regarding interpretability extend to the spatial dimension as well, as a correction for multiple comparisons would be computed based on two-dimensional cluster extent across phase x channel. We hope that the code we provide on our GitHub repository for both Matlab and Python applications can be flexibly adapted and extended to answer a wide variety of research questions in the field today.

## Supporting information

S1 Fig Probability of reporting an existing effect for the entire simulated parameter space. **a**, Probability of reporting an existing effect (Type II Error) as a function of the Family Wise Error Correction (FWER) method (circular clustering vs Omnibus), the statistic adopted (t-value or Robust), and the intersubject noise level simulated (natural log). **b**, Probability of reporting an existing effect as a function of the FWER method, the statistic adopted, and the trial noise level simulated (natural log).
(DOCX)

S1 File Berther et al. (DOCX)
