# Supplementary material for: Robust circular cluster-based statistics for respiration-brain coupling
Source: PLoS Comput Biol. 2026 Sep 3;22(9):e1014672. doi: 10.1371/journal.pcbi.1014672 (PMC13541119; doi:10.1371/journal.pcbi.1014672)
Supplement: Supplementary file 4 [file pcbi.1014672.s002.docx]

**Supplementary Information**

**Simulation rationale, model and parameter space**

To validate our method of statistical circular clustering, we conducted a simulation to assess the probability of both Type I and II errors (false positive and false negative, respectively) of our approach.

We used the following formula to model probability of responding to a stimulus as a function of the respiratory phase:

$$p_{raw}= effect*cos(\theta_{trial} +{Noise}_{intersubject})+ Noise_{trial}$$

Where $effect$ codes for the magnitude of the experimental effect, $\theta_{trial}$ to the respiration phase angle at the single trial, ${Noise}_{intersubjects}$ to the phase lag with respect to 0, constant within the subject but introducing lack of coherence across subjects, and $Noise_{trial}$ as random variability in the probability of response at the single trial level. Both noise parameters were sampled from a gaussian distribution, whose standard deviation was scaled for different noise levels (detailed below).

In order to maintain the final probability of response $p_{resp}$ within reasonable ranges, i.e. to trim ${p_{raw}<0.01}$and ${p_{raw}>0.99}$, the following clamping functions were applied:

$$p_{resp} = min(0.99, max(0.01, p_{raw}))$$

The ${p_{resp}}$above was used to determine, by mean of a binomial distribution, the outcome of every single trial, for ${n_{trials}}$and for $n_{subjects}$.

The parameter space was thought to provide an adequate granularity for those variables of interest that could change the experiment outcome, i.e. whether an experimental effect would be reported. These variables include the already mentioned $effect$, $n_{trials}$, $n_{subjects}$ , ${Noise}_{intersubjects}$, ${Noise}_{trial}$, but also analytical choices, such as the width of the bins used, the number of phase bins, the statistic used to evaluate the presence of an effect in each phase bin, and the method to correct for multiple comparisons.

We thus included:

*i)* Thirty-one values of effect magnitude, linearly spaced from 0 to 0.3.

*ii)* Three values for subjects number, 10, 20, 30.

*iii)* Five values for trials number, 100, 200, 300, 500, 800.

*iv)* Seven values of ${Noise}_{trial}$, logarithmically spaced between $exp(-3)$ and 1.

*v)* Seven values of ${Noise}_{intersubjects}$, logarithmically spaced between $exp(-3)$ and 1.

*vi)* Three values of binning width, $2\pi/20,2\pi/10, 2\pi/5$.

*vii)* Four values of number of phase bins, 30, 45, 60, 75.

*viii)* Two possible measures of relative dispersion: t values and median/iqr, the latter being a measure used consistently for a more robust scaling against outliers [45-46].

*ix)* Two possible family-wise error rate (FWER) correction methods: our circular clustering method proposed here and the omnibus correction [47], consistently adopted for multiple corrections in studies involving dense sampling of behavioral responses [48-52]. In brief, given a set of statistics of size $n_{bins}$times $n_{permutations}$, the maximum (or minimum) value over all the bins is taken for each permutation. This set, of size $n_{permutations}$, is taken as the surrogate set to define the upper (or lower) boundaries for significance of the empirical statistic.

The combination of all the aforementioned parameters lead to 1093680 scenarios, each tested 20 times for consistency.

Each combination was considered to have found an existing result (true positive) if it would find at least one significant datapoint of an existing effect. Positive results given absence of effect would be classified as Type I error (false positive).

The simulation was programmed in python 3.13. The single core functions of the simulation were optimized with *numba* 0.63.1 and the parallel execution over the High Performance Cluster was handled via HyperQueue 0.24.0.

**Supplemental Figure 1. Probability of reporting an existing effect for the entire simulated parameter space.** **a**, Probability of reporting an existing effect (Type II Error) as a function of the Family Wise Error Correction (FWER) method (circular clustering vs Omnibus), the statistic adopted (t-value or Robust), and the intersubject noise level simulated (natural log). **b**, Probability of reporting an existing effect as a function of the FWER method, the statistic adopted, and the trial noise level simulated (natural log).

**References**

45.	Pedregosa, F., Varoquaux, G., Gramfort, A., Michel, V., Thirion, B., Grisel, O., ... & Duchesnay, É. (2011). Scikit-learn: Machine learning in Python. the Journal of machine Learning research, 12, 2825-2830.

46.	Balestrieri, E., Chalas, N., Stier, C., Fehring, J., Gil Ávila, C., Dannlowski, U., ... & Gross, J. (2025). Beyond oscillations - Toward a richer characterization of brain states. Imaging Neuroscience, 3, imag_a_00499. https://doi.org/10.1162/imag_a_00499

47.	Nichols, T. E., & Holmes, A. P. (2002). Nonparametric permutation tests for functional neuroimaging: a primer with examples. Human brain mapping, 15(1), 1-25. https://doi.org/10.1002/hbm

48.	Michel, R., & Busch, N. A. (2023). No evidence for rhythmic sampling in inhibition of return. Attention, Perception, & Psychophysics, 85(6), 2111-2121. https://doi.org/10.3758/s13414-023-02745-x

49.	Re, D., Inbar, M., Richter, C. G., & Landau, A. N. (2019). Feature-based attention samples stimuli rhythmically. Current Biology, 29(4), 693-699.: doi:10.1016/j.cub.2019.01.010

50.	Song, K., Meng, M., Chen, L., Zhou, K., & Luo, H. (2014). Behavioral oscillations in attention: rhythmic α pulses mediated through θ band. J Neurosci. 34(14), 4837-4844. https://doi.org/10.1523/JNEUROSCI.4856-13.2014

51.	Balestrieri E, Ronconi L, Melcher D. Shared resources between visual attention and visual working memory are allocated through rhythmic sampling. Eur J Neurosci. 2022;55(11–12): 3040–3053. https://doi.org/10.1111/ejn.15264

52.	Huang, Y., Chen, L., & Luo, H. (2015). Behavioral oscillation in priming: competing perceptual predictions conveyed in alternating theta-band rhythms. J Neurosci. 35(6), 2830-2837. https://doi.org/10.1523/JNEUROSCI.4294-14.2015
