# Supplementary material for: Robust circular cluster-based statistics for respiration-brain coupling
Source: PLoS Comput Biol. 2026 Sep 3;22(9):e1014672. doi: 10.1371/journal.pcbi.1014672 (PMC13541119; doi:10.1371/journal.pcbi.1014672)
Supplement: Supplementary file 10003 [file pcbi.1014672.s003.pdf]

Response to Reviewers

We would like to thank all reviewers for their positive feedback and constructive comments on our manuscript. Main changes made in response to the reviewer's comments are:

- Addition of a comprehensive simulation analysis underscoring good control of both Type I and II errors
- Addition of a more detailed description of key concepts and parameters
- Addition of a step-by-step markdown file outlining the practicalities of our pipeline
- Revision of all figures

In what follows, we address all comments in a point-by-point reply. Reviewer comments are shown in **grey**, our responses in **blue**, and quoted changes to the manuscript in **red**.

Reviewer #1: This manuscript presents a methodological tutorial and code-supported workflow for respiration phase analyses and respiration–brain coupling. The pipeline combines (i) respiratory phase extraction, emphasising landmark-based interpolation, (ii) surrogate generation, with a focus on iterated amplitude-adjusted Fourier transform (IAAFT) surrogates, and (iii) circular cluster-based permutation testing to address multiple comparisons while respecting wrap-around adjacency at phase boundaries. The authors aim to improve reproducibility and inferential validity in the respiration phase coupling literature.

The contribution is timely and likely to be useful for researchers working on respiration/body/brain interactions. The manuscript is well motivated and practically oriented. However, some elements would benefit from clearer formalisation, additional validation or methodological detail, and more explicit decision rules, particularly where analytical degrees of freedom may affect outcomes. The main points are as follows.

We appreciate the reviewer's positive assessment of our manuscript and respond to their comments in detail below.

<u>Major comments:</u>

1. Clarify the binning and windowing parameterisation (k, ω, overlap, coverage): The binning scheme is central to the workflow and to the claim that parameter choices influence sensitivity and power. However, the mapping between the number of bins (k), bin width (ω), overlap, and full-cycle coverage is not sufficiently explicit. In particular, small k settings are difficult to interpret when ω is fixed. It would be helpful to provide a concise formal specification of the scheme, including: (i) how bin centres are placed as a function of k, (ii) whether each bin is a sliding window of width ω centred on each bin centre, (iii) how overlap is defined, and (iv) how wrap-around membership is handled near ±π. A short worked example for small k would likely resolve ambiguity.

Thank you for prompting us to provide additional clarification on a few core parameters for phase binning. The relationship between number of bins and bin width to cycle coverage is

rather straightforward, with the overlap across windows being a logical consequence: First, the width of the moving window (ω) is defined only once and then kept constant. Second, the analyst decides on the number of phase bins (k), i.e. how fine the resolution of the outcome variable across the respiratory cycle ought to be. For a clearer illustration of the approach, we consider a medium-resolution example rather than a small k here, and compute hit rates for k = 30 phase bins across the cycle with a moving window width of ω = 2π/10 = 36° (as shown in Fig. 2d of the manuscript). In this case, we start by computing the hit rate across all trials presented e.g. at respiratory phases ranging from 0° ± ω/2, i.e. -18° to 18° and assign that value to the bin centre at 0°. Then we move forward in steps of Δφ = 360°/k = 360°/30 = 12°, apply the same moving window with a width of ω = 36° (this time reaching from 12° ± 18°, i.e. from -6° to 30°), compute the hit rate within this subset of trials, and assign this value to the bin centre at 12°. This procedure is repeated k = 30 times until the entire respiratory cycle has been covered.

During this procedure, the overlap between neighbouring phase bins results from the relationship between the number of bins (k) and the width of the moving window (ω): In the above example, we cover the respiratory cycle in steps of Δφ = 12° and apply a moving window of ω = 36°, i.e. ω/2 = 18°. Since the moving window is defined symmetrically around the center of each phase bin, it follows for each combination of k and ω that whenever Δφ < ω/2, the incremental steps of the moving window are smaller than half its width, which results in overlap. For Δφ = ω/2, neighbouring phase bins would be exactly aligned, while Δφ > ω/2 will likely lead to the adverse scenario that e.g. some trials/responses are not considered in the analysis at all (since the moving window will not cover all phase angles).

For clarity, the manuscript has been revised as follows (l. 265):

> For the phase binning approach, the analyst defines i) the width of the moving window (ω) as well as the number of phase bins used (k; see Fig. 2c). Together, these parameters determine how fine the resolution of the outcome variable across the respiratory cycle ought to be. As a practical example, imagine that hit rates ought to be computed for k = 30 phase bins across the cycle with a moving window width of ω = 2π/10 = 36°. In an iterative approach, hit rate is first computed across all trials presented at respiratory phases ranging from 0° ± ω/2, i.e. -18° to 18°, and assigned to the bin centre at 0°. The analyst then moves forward in steps of Δφ = 360°/k = 360°/30 = 12°, applies the same window width of ω = 36° (this time reaching from 12° ± 18°, i.e. from -6° to 30°), computes the hit rate within this subset of trials, and assigns this value to the bin centre at 12°. This procedure is repeated k = 30 times until the entire respiratory cycle has been covered (see Fig. 1c).
>
> During this procedure, the overlap between neighbouring phase bins results from the relationship between the number of bins (k) and the width of the moving window (ω): Since the moving window is defined symmetrically around the center of each phase bin, it follows for each combination of k and ω that whenever Δφ < ω/2, the incremental steps of the moving window are smaller than half its width, which results in overlap. For Δφ = ω/2, neighbouring phase bins are contiguous – meaning their boundaries meet without overlap, while Δφ > ω/2 will likely lead to the adverse scenario that some trials are not considered in the analysis at all (since the moving window will not cover all phase angles).

In addition, for a better illustration of the phase binning procedure (ω and overlap in particular), we have updated Fig. 1c as follows:

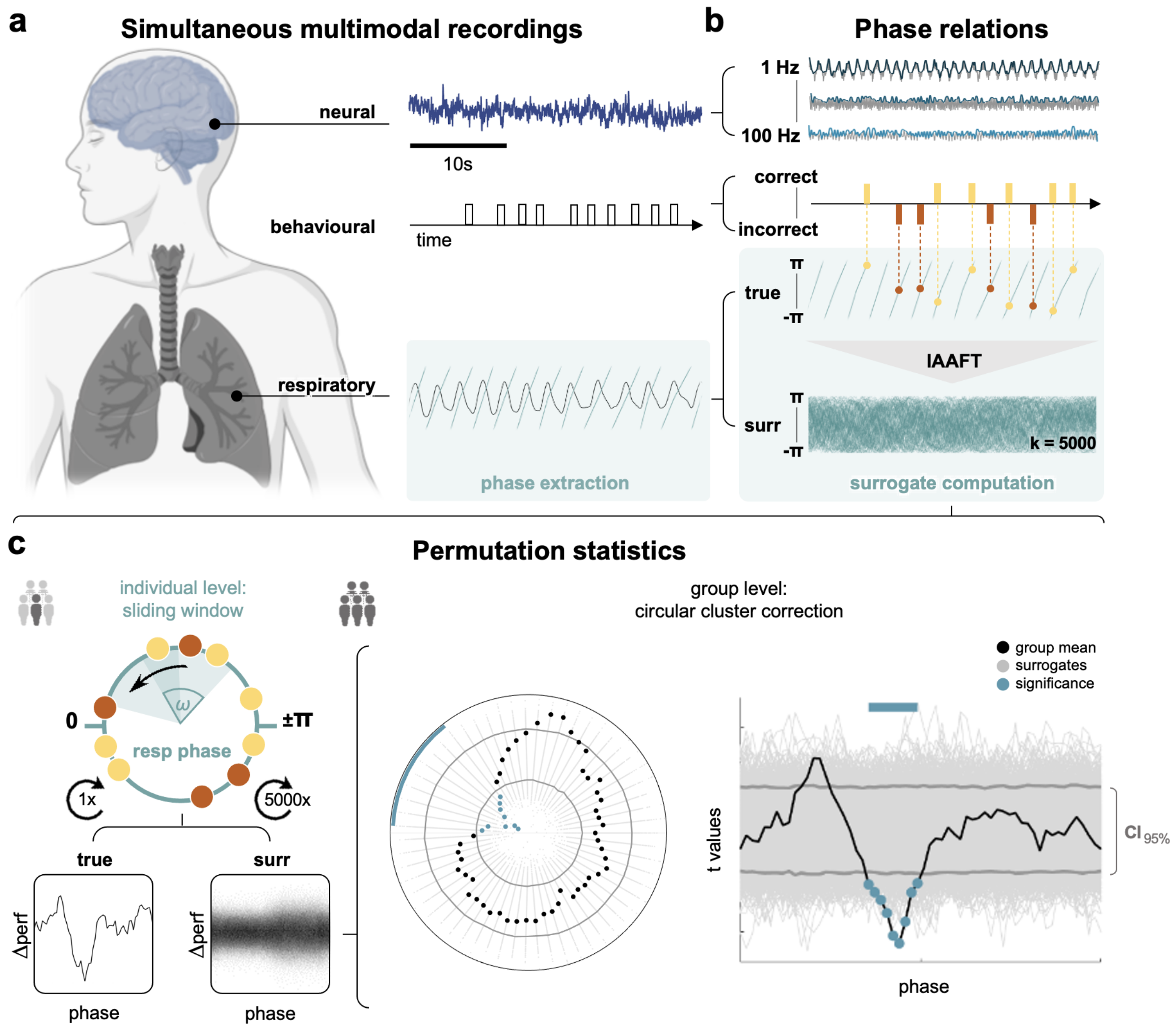

Kindly note that examples with a very low number of phase bins (k = 2 and k = 4, respectively) are shown in Fig. 2d. The corresponding analysis of hit rate ~ respiration phase with such few phase bins clearly shows the lack of sensitivity compared to solutions with a higher number of phase bins (e.g., k = 30).

2. Specify a bit more a reproducible protocol for parameter tuning on an independent subset: The manuscript recommends selecting binning parameters on an independent subset to mitigate overfitting and improve power, but the practical procedure and selection criterion are underspecified. A simple split-sample or cross-validation protocol, paired with an objective criterion, would reduce subjectivity. For example, parameters could be chosen to stabilise an a priori summary metric while maintaining a minimum number of events per bin per participant.

We appreciate the suggestion of a more objective assessment during parameter selection. We have amended the manuscript and recommend a split-sample protocol with a pilot sample of 10-20% of the desired sample size for parameter selection. Based on the analyses

exemplified in Fig. 2d, the investigator can then make informed decisions, e.g. regarding the number of phase bins. The revised manuscript reads as follows (l. 287):

> Ideally, to answer these questions, analysts should use independent data to systematically vary parameters like the number and width of phase bins in order to identify those which yield the highest statistical power. This combination of parameters can then be applied in the analysis of the main dataset. As a general guideline, we suggest parameter selection in either pilot data or an independent subset with a sample size of around 10-20% of the desired final sample.

3. Phase definition under breath holds and plateaus requires more explicit, physiology-informed rules: The tutorial motivates landmark-based interpolation using local maxima and minima, yet transition detection can be ambiguous in belt-based respiration (and other modalities) when breath holds or plateau-like segments occur near the expiratory trough. Choosing an earlier versus later minimum can extend one phase at the expense of the other. This matters because events near boundaries may be reassigned to different phases, potentially affecting estimates of phase locking and phase-resolved effects.

Relatedly, onset-focused alternatives, including trapezium-area approaches (Esra al.; Galvez-Pol et al. ; Engelen et al...) and slope or derivative-based onset detection, may better capture physiologically plausible transitions in some contexts. A physiology-informed choice would ideally yield phase durations consistent with typical breathing dynamics, rather than allowing signal artefacts to systematically stretch or compress phases across participants or tasks. I recommend (i) stating an explicit rule for trough selection under plateaus (for example, first versus last candidate minimum, or a slope-threshold criterion), (ii) justifying this choice with respect to respiratory physiology and measurement modality, and (iii) including a brief sensitivity analysis comparing an extremum-based approach with an onset-based alternative.

Thank you for raising the issue of meaningful/realistic interpretation of respiratory phase. In response to these suggestions, we have added the trapezoid approach to the comparison of phase extraction methods shown in Fig. 2a:

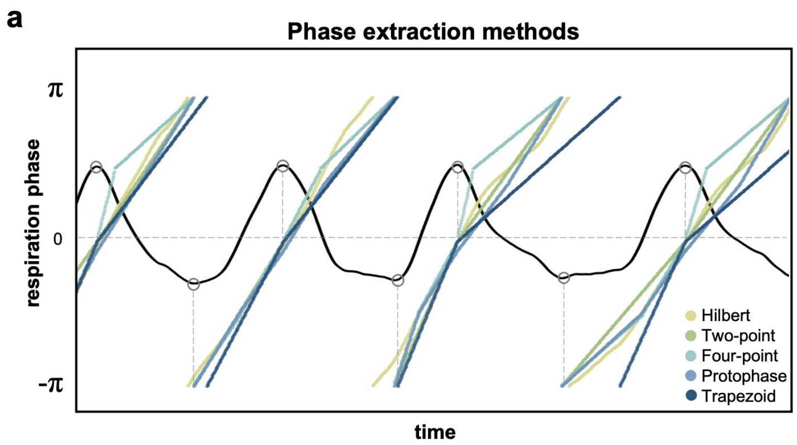

Moreover, the following figure demonstrates that group-level results remain stable and virtually identical for both the trapezoid and two-point interpolation of respiratory phase:

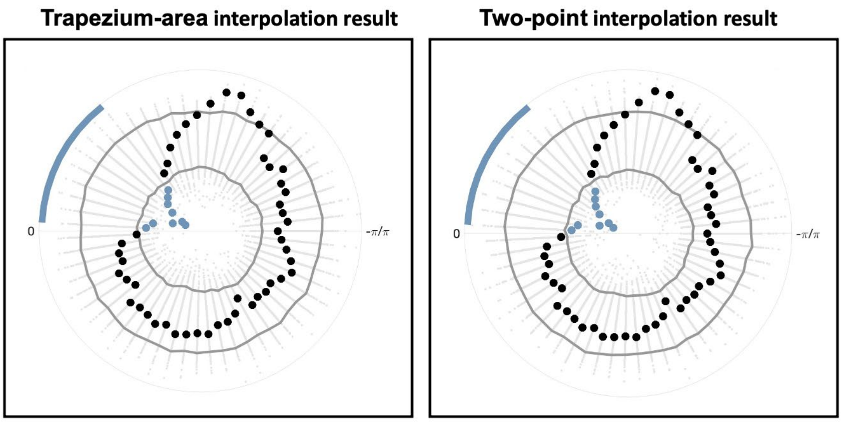

As an additional remark, we would like to emphasize that the extent to which these rather minor changes in respiratory phase (e.g. trough definition during a plateau) affect final results will critically depend on the aforementioned analysis parameters: Both a relatively wide moving window and/or a large amount of overlap between steps of the moving window will cause 'misplaced' trials to be smoothed as an outlier across many trials within the same phase bin.

Finally, the interpolation approaches we describe in our pipeline take the absolute lowest point as the onset of a respiratory cycle. This is due to the use of respiration belt transducers in our example data, which matches the most commonly used recording method for respiratory signals in the field today. Here, the absolute minimum indicates peak expiration (i.e. the lowest amount of air in the lungs). A comment regarding this has been added to the manuscript (l. 217):

> In our respiratory belt examples, the absolute minimum identifies peak expiration, i.e. the lowest amount of air in the lungs before the next inspiratory phase.

4. Provide additional validation of the circular cluster-based procedure: Readers may expect explicit evidence that the proposed procedure controls family-wise error under realistic null scenarios and has adequate power across effect widths and noise structures. A concise simulation-based validation, in the main text or supplement, varying sample size, effect width, event non-uniformity across phase, and noise characteristics, would strengthen confidence. A benchmark comparison to a simpler correction strategy (for example, max-statistic across bins) would further contextualise the method.

We appreciate the reviewer's suggestion to further substantiate our method proposal by adding a simulation analysis (which was echoed by Reviewer #2). Following both remarks, the revised manuscript now includes a comprehensive simulation section. Compared to an approach using the Omnibus correction, our circular clustering test yielded equal or better probabilities of reporting an existing effect across different levels of noise in effect phase coherence as well as single trial response variability (see newly added Fig, 4a+b in the revised manuscript). Though less conservative than the max-statistic across bins, our approach still keeps the Type I Error at the expected level of 0.05 (cf. Fig. 4c). With the simulation, we also provide an assessment of statistical power across different experimental and preprocessing choices including sample size (newly added Fig. 5 in the revised manuscript). Simulation

results and figures can be found in the main text of the revised manuscript in the section 'Simulation-based validation of the circular cluster-permutation procedure' (starting in line 497). We have additionally added further details on the simulation rationale, model, and description of parameter space as well as an additional figure providing an overview of Type II Error rates across all simulated noise levels and characteristics in the supplementary material.

5. The manuscript notes differences between respiration and other rhythms, but it would be useful to delineate which components generalise beyond respiration. A short scope statement could distinguish: (i) general circular inference tools (for example, circular cluster correction), (ii) respiration-optimised phase extraction guidance, and (iii) conditions under which alternative surrogate or phase-definition strategies are preferable.

Thank you, we have refined the previous scope statement to more clearly delineate which functionalities generalize to other applications. The revised manuscript now reads as follows (l. 657):

> Rather, it provides a general statistical framework for the analysis of behavioural or neural data in relation to any circular signal (e.g. recorded across respiratory phase, visual angles, or time of day) based on established practices in the field of neuroscience. As we note at the outset, however, the properties of physiological signals recorded from the heart, lungs, and stomach, are very diverse with regard to their respective frequencies. As a consequence, the systematic comparison of phase extraction methods was conducted specifically for respiratory analyses and will not hold for cardiac data (which are discretised into diastole and systole) or gastric data (which can usually be handled with a simple Hilbert transform). Moreover, the proposed IAAFT-based approach will likely not be beneficial for gastric recordings, for example: The low frequency combined with high regularity of the signal means that the IAAFT algorithm will have a hard time generating adequate surrogates whose phase is not highly correlated with that of the original signal.

<u>Minor comments:</u>

i) Terminology: Use “bin”, “window”, and “overlap” consistently, and/or define “quasi-continuous” early and precisely.

Thank you, the revised manuscript uses consistent terminology and we have added an early definition of ‘quasi-continuous’ (l. 143):

> Particularly for a high number of phase bins, we refer to these signals as ‘quasi-continuous’, i.e. so densely sampled across the respiratory cycle that they approximate continuous phase (in contrast to a discrete inspiration vs expiration distinction).

ii) Implementation details: Report default IAAFT settings, convergence checks, and approximate computational costs for typical signal lengths.

We appreciate the suggestion and refer the reviewer to the documentation in the GitHub repository for specific implementation details, as these details are subject to possible future change but cannot be updated in the published manuscript. Based on another reviewer's request, we have added a markdown file with step-by-step instructions and references to the code that also includes instructions on IAAFT parameters and convergence checks, as well as reporting some benchmarks on computational costs for different signal lengths.

iii) Reporting guidance: Provide a brief reporting template specifying the phase-definition method, surrogate type, and correction method to support standardised reporting across studies.

The reviewer's suggestion is much appreciated and we have added suggestions for standardised reporting as follows (l. 648):

> Given the demonstrably strong influence of methodological choices outlined in this paper, we further suggest to clearly report key parameter choices in studies of neural or behavioural data as a function of physiological signalling. At minimum, these should include the method of phase extraction (with exemplary visualisation), phase binning (with detailed information regarding bin number and width), surrogate generation, and correction for multiple comparisons.

Reviewer #2: Signed review: Guillaume Rousselet

This is a very interesting and useful article. You make a lot of important points, which are well explained in the main text and well summarised at the end. I haven't tried the code but the GitHub repository is well organised and documented. There two main points that you should address, and a few minor points to consider about the language.

Thank you for the positive overall assessment of our manuscript and please find point-by-point responses below.

Main points:

The main issue is the lack of validation. Could you provide a simulation that demonstrates false positive rates and power for a few realistic cases? All new methods must be supported by reproducible simulations. The examples and rationale you provide are compelling, but not sufficient.

We thank the reviewer for giving us the opportunity to strengthen our approach by means of simulation data. Since a similar point was previously raised by Reviewer 1, we reiterate the main results of our new simulation example here: Type I Error rates of our circular cluster permutation test remained below the expected level of 0.05, with equal or better detection performance of an existing effect across different noise levels and characteristics than a more classical max-statistic FWER correction. Additionally, we now provide an estimate of statistical power across a range of experimental and preprocessing choices, including parameters discussed in the manuscript (e.g. number of bins and bin width) as well as more fundamental design variables such as sample size and number of trials. Simulation results and their visualization can be found in the main text of the revised manuscript (l. 496ff), and further details on simulation model and parameter space are provided in the supplement, with accompanying code in the GitHub repository.

The second main point is about accessibility: in all figures, please increase the contrast and use colour-blind friendly colour palettes. In figure 3b, there is too much empty space and the colours are superfluous.

Panels in Figure 1 are too small. In figure 1c bottom panel, please remove the little stars. P and t values do not measure effect sizes, importance or reliability. A horizontal line marking statistical significance is sufficient.

Thank you for prompting us to make our figures more accessible. As a result, all figures have been revised following the reviewer's suggestions (see revised manuscript).

Minor points:

Robust statistics are mentioned in the abstract but not explain, in particular in relation to the specific issues in the literature. In other words, robust to what?
What are the particular statistical issues in your field? The introduction does a good job at summarising the issues with the statistical treatment of various measurements. The abstract

should summarise these issues to motivate the work explicitly.

Thank you for this pointer, the Abstract has been revised and now reads as follows:

> This quest for robust methods is further complicated by the (naturally) circular data involved in the study of phase-locked effects, e.g. in respiration-brain coupling. Various available approaches for phase extraction, constructing adequate surrogate data for statistical comparison, and accounting for the circularity of respiratory data lead to poor cross-study generalisability of results. Robustness of effects is particularly affected by the problem of multiple comparisons in phase-related inferential statistics.

In the introduction, there is a lot of important points about binning. It would be worth pointing out the broader literature about the negative consequences of dichotomisation of continuous variables -- see for instance:

MacCallum RC, Zhang S, Preacher KJ, Rucker DD. On the practice of dichotomization of quantitative variables. Psychol Methods. 2002 Mar;7(1):19-40. doi: 10.1037/1082-989x.7.1.19

The cost of dichotomising continuous variables
https://www.ncbi.nlm.nih.gov/pmc/articles/PMC1458573/

In addition to Maris & Oostenveld (2007) you should cite the more directly relevant Maris, E., Schoffelen, J.-M., & Fries, P. (2007). Nonparametric statistical testing of coherence differences. Journal of Neuroscience Methods, 163(1), 161–175. https://doi.org/10.1016/j.jneumeth.2007.02.011

We appreciate the suggested references and have included them in our revised manuscript.

Also explain how your work relates to their effort dealing with phase coherence. In that context, is your method of computation of surrogate distributions really a permutation test? Typically, permutation relies on exchangeability between independent conditions. Here it seems you are approaching the data differently. Would it be more appropriate to describe your method as random shuffling without using the term permutation?

"Conventional cluster-based permutation tests assuming a non-circular independent variable fail to account for the wrap-around continuity inherent to circular data." -- but this is precisely because standard permutation tests address a different question, involving two independent groups, the assumption of exchangeability, to test whether the two distributions have identical shapes. Your approach addresses a different question.

We fully agree that the question of exchangeability between empirical and surrogate data constitutes the foundation of cluster permutation testing, which is precisely why our approach is indeed a permutation procedure: As with conventional continuous applications, we also test against exchangeability between different distributions - how we arrive at adequate surrogates for these tests is one of the core points of our paper. Also note that permutation testing is of

course available e.g. for dependent-sample t-tests or repeated-measures ANOVAs, which is simply to say that the general principle of using permutations (in our case, IAAFT-derived phase surrogates) is by no means limited to specific applications or questions.

Why t-test on means, given that sample means and sample SD are not robust? Could be trimmed means if you want to achieve robustness against outliers and skewness.

We thank the reviewer for this important question. In principle, robustness to outliers and skewness can be considered both at the single-subject level during phase-binning using the moving window and at the group level during the computation of summary statistics. In our example application, the trial-wise outcome is binary (hit vs. miss) with phase-bin means representing hit rates, thus trimmed means at the trial level are not informative. For other applications in which non-binary continuous trial-wise outcome measures are aggregated within phase bins, the use of trimmed means may well be appropriate and beneficial. For group-level inference, our cluster-based procedure uses mean/SD-derived t-values as a summary statistic across subjects, but statistical significance is evaluated against a permutation-derived null distribution rather than the theoretical t-distribution. Thus, inference does not rely on the parametric assumptions of a conventional t-test to the same extent, with the permutation framework inherently providing more robust statistical inference. We further chose t-values as the default summary statistic because they are widely used, readily interpretable, and account for sample size. The robustness of our proposed approach is further illustrated by the additional simulations performed at the request of Reviewers #1 and #2: relative to the approach based on robust scaling using the interquartile range, the use of t-values as a measure of relative dispersion performed at least as well (and in some cases better) across all levels of intersubject and single-trial noise, for both Type I and Type II error rates. However, we also provide the simulation code implementing interquartile-range scaling, so that users may adopt this approach if preferred or required for their application.

"t-tests and ANOVAs or their respective non-parametrical equivalents" -- in general, non-parametric models, such as rank-based models, tests different hypotheses than ANOVAs. To avoid that issue, and since a t-test is an ANOVA, you could write something like "parametric or non-parametric models".

The suggested phrasing was added to the manuscript (l. 64).

"Phase-amplitude coupling means that the phase, for example that of the respiratory rhythm, influences the amplitude of neural activity" -- is the causal language appropriate here or could you rephrase in terms of association?

The sentence has been rephrased (l. 134):

> Phase-amplitude coupling means that the phase, for example that of the respiratory rhythm, systematically covaries with the amplitude of neural activity, e.g. in the alpha frequency band.

"chance level" is mentioned several times. Could you rephrase to avoid readers thinking wrongly that NHST can determine if an effect is due to chance? This is not what you wrote, but we're one step away from an incorrect and common interpretation. Something like "larger

than the effect from a random partition of the data" in the case of a permutation test. See more careful descriptions in Maris et al. (2007).

The suggested phrasing was added to the manuscript (l. 82, l. 149).

"Reliable extraction of respiratory phase" -- is reliability, in the sense of test-retest reliability, the appropriate term here? This section seems to be about accuracy instead.

The term has been replaced as suggested (l. 121, l. 210).

Reviewer #3:

This manuscript presents a methodological and tutorial-style framework for studying respiration–brain coupling, focusing on (i) respiratory phase extraction, (ii) surrogate data generation (with emphasis on IAAFT methods), and (iii) a circular extension of cluster-based permutation statistics to address multiple comparisons in phase-binned analyses. The topic is timely and relevant for the growing interest in brain–body interactions and interoceptive neuroscience. The proposed circular cluster-based inference is potentially valuable.

However, the manuscript currently lacks clear conceptual positioning, explicit novelty delineation, and a fully transparent analysis pipeline. Several aspects are presented in an oversimplified manner, particularly regarding respiratory signal features. Overall, the work would benefit from substantial clarification and restructuring to strengthen both scientific rigor and tutorial usability.

I therefore recommend major revision.

1) The manuscript discusses respiration–brain coupling within “brain-body neuroscience” and interoception frameworks but does not explicitly situate the work within the established network physiology paradigm, which has already introduced a systems-level view of interacting organ networks [1]. Moreover, the authors do not clearly contrast their approach with classical multimodal pipelines commonly used in the literature—such as extracting EEG spectral content by frequency band and relating it to autonomic indices (e.g., sympathovagal balance derived from HRV metrics computed from ECG).

We thank the reviewer for their remarks and would like to respond to both points separately. First, regarding a network physiology view of interactions across organ systems, it is important to note that the present methodological paper does not aim at all to provide a conceptual framework of respiration-brain coupling, but to introduce a step-by-step pipeline for data processing and a novel statistical approach to account for circularity of phase data. As e.g. noted by Reviewer 1, this circular cluster permutation approach is by no means limited to physiological or brain-body contexts, which is why we explicitly do not discuss respiration-brain coupling in any greater conceptualisation of physiology (but have done so extensively in previous work).

Second, as for the distinction between our approach and cardiac analyses such as spectral power across variation in HRV, we explicitly point out the particulars of cardiac, respiratory, and gastric data in a dedicated section (l. 59 ff.):

> Questions involving the cardiac rhythm, for example, are commonly reduced to comparisons of neural or behavioural data during systole vs diastole, although this distinction comes with its caveats regarding the actual onset definitions of systole and diastole [13]. Since the ECG is a relatively fast signal which cannot be consciously modulated directly, phase-related analyses going beyond the widely accepted systole/diastole dichotomy are very rare. From a statistical point of view, this considerably simplifies analyses to parametric or non-parametric models. While such simplifications come with no obvious disadvantages in the specific case of cardiac-

based analyses, they fall short of adequately representing physiological signals which are either slower in rhythmicity (gastric) and/or under voluntary control (respiration).

We continue this argument later on by pointing out the specific requirements of respiratory phase analysis (and how the field so far has been unable to address them; see l. 88 ff):

> Finally, analyses of respiration-brain coupling constitute a special case for several reasons: Most prominently, respiration is the only physiological rhythm which is fully under voluntary control. [...] This is why, despite its continued popularity, discretizing the respiratory signal into a mere contrast of 'inspiration vs expiration' does not adequately represent the rich and variable dynamics of the signal (for general critiques of discretizing continuous data, see [17,18]). This is further highlighted by consistent reports of respiration phase-dependent effects during transition times (e.g. from inspiration to expiration and vice versa [19,20],24]) or other specific phases [16],[19],[9,21–23] such as the strongest changes in airflow (i.e. the inflection point of the respiratory signal). Simply collapsing continuous respiratory phase into two discrete 'states' would make the investigator blind to detecting such momentary modulations and drastically reduces the sensitivity of respiration-related analyses. Rather, the full information from continuous respiratory phase should be exploited to maximise signal-to-noise ratio.
> As mentioned at the outset, the field's hesitancy to implement rigorous analyses of continuous respiratory phase may at least partly be explained by the absence of validated statistical approaches. Two things are needed: A reliable approach for continuous, artefact-free phase extraction, and inferential statistics with adequate control over false positive results and multiple comparison correction. [...] On the other hand, while cluster-based permutation correction [24,25] has become the gold standard for neuroscientific analyses of linear data (such as time series or evoked responses), an equivalent solution for circular data is currently not available.

Critically, the linear (i.e. non-circular) data format referred to in the quoted passage includes exactly the type of analyses mentioned in the reviewer's comment: Relating spectral M/EEG power to a physiological parameter like sympathovagal balance will ultimately be an analysis of two time series (HRV ~ time, power ~ time). Since neither of these signals is circular in nature, conventional statistics are well-suited to answer questions regarding the relationship of these two time series. This is not the case for respiratory phase ~ time, which is why we strongly contrast our novel approach with the properties of other organ systems.

The final argument to this effect comes in the concluding section of the manuscript and in the tradition of 'Ten simple rules' papers, where we highlight the most crucial take-home aspects of our work. Here, 'rule' #2 (*Be mindful of domain-specific caveats that come with analysing cross-frequency brain-body data*), #7 (*Collapsing continuous respiratory phase into an inspiration vs expiration dichotomy will almost always be the less sensitive [...] choice*), and #10 (*Make sure to account for the circular nature of phase data and avoid tests or corrections which assume linear data*) all specifically outline the specific aspects in which phase-related analyses of respiratory data are different from other organ systems.

2) Several statements implicitly treat respiratory phase as the primary or exclusive meaningful descriptor of respiration–brain interactions. This is an oversimplification. There is substantial

literature demonstrating the importance of respiratory amplitude, volume, and breath-by-breath variability, including methods extracting respiratory amplitude aligned to cardiac R-peaks and analyzing its variability.

3) The manuscript employs phase–amplitude coupling where respiratory phase modulates neural amplitude. However, the rationale for privileging phase over other respiratory descriptors is not sufficiently justified. it would be equally informative to study modulation driven by respiratory amplitude dynamics, which may capture different physiological mechanisms.

Since these two comments touch upon a very similar point (i.e. the present focus on respiratory phase), we respond to them jointly here. We appreciate the opportunity to clarify the prioritisation of phase over other parameters like depth/amplitude or variability. We wholeheartedly agree with the Reviewer's assessment that these other parameters are of great interest to gain a substantial understanding of respiration-brain coupling. This is why, in previous empirical work, we have conducted in-depth analyses of respiratory hallmarks like breathing depth (Kluger & Gross, NeuroImage 2020), nasal vs oral breathing (Kluger et al., Nature Communications 2023), and respiratory variability (Chalas et al., Nature Communications, in press). Furthermore, these parameters are one core focus of an ongoing ERC Starting Grant dedicated to illuminating the higher-order interplay of precisely these parameters.
Statistically, these applications pose a much simpler challenge: Within-subject effects of deep vs normal breathing, for example, can be quantified using a simple t-test; continuous parameters like breath-to-breath variability can be included in an equally simple linear regression model. The special case of respiratory phase analyses has so far been more difficult to handle due to the circular nature of phase data. Hence, the core novelty of our tutorial here was to provide a robust statistical pipeline for reliable and sensitive statistics on these circular data (kindly also see our response to the next comment).

In order to avoid our present manuscript from being misunderstood as an argument against the importance of other respiratory parameters, we have amended the main text to i) stress the field's interest in these parameters and ii) outline why the present paper is solely focussed on respiratory phase (l. 44):

> Analyses of physiological parameters like breathing depth [7,8], pathways [9,10], or respiratory rate [11,12] can be conducted with straightforward statistical comparisons across two or more conditions. Phase-locked analyses of physiological rhythms, on the other hand, are not only lacking such a contrast condition, but phase data is inherently circular and therefore do not allow for a convenient parameterization of the underlying distribution. Hence, statistical approaches currently being used to circumvent these difficulties are rather heterogeneous, which is why the present tutorial paper focuses on analyses of continuous phase.

4) A significant portion of the manuscript reads as a methodological overview of known problems (e.g., surrogate limitations, multiple-comparison issues), but it is not always clear what constitutes the novel contribution of the present work. At present, the main innovation

appears to be: the circular cluster-based permutation statistic and its integration into a recommended pipeline. However, this is not explicitly framed.

The Reviewer precisely points to the key novel aspects of the present manuscript, namely the development of circular cluster-correction statistics and its embedding in a step-by-step tutorial pipeline. In response to the Reviewer's remark, we have revised the manuscript in different instances to more clearly outline the novelty of our work:

> In this tutorial, we propose a robust pipeline for respiration phase-related analyses based on a novel circular extension of cluster-based permutation testing. (l. 26)
>
> Therefore, in what follows, we describe an easy-to-use pipeline for continuous phase extraction and the joint analysis of respiratory and neural or behavioural data and introduce a novel extension of widely used cluster permutation testing for circular data. (l. 114)
>
> Second, we present a novel extension of cluster permutation procedures specifically for circular data in order to reliably control error rates in phase-related analyses. (l. 584)

5) Despite the presence of a methods synopsis and schematic figure, the manuscript does not provide a sufficiently explicit step-by-step workflow. As a result, it remains unclear how a reader should proceed starting from raw EEG (or MEG) signals to final statistical inference. I recommend also to better explain the flow of the paragraph named "Nexus 3: A novel approach for circular cluster-based permutation testing"

We appreciate and share the Reviewer's call for a step-by-step workflow which was one of the key aims motivating our manuscript. Importantly, however, our tutorial was not intended to be focussed on the processing of M/EEG data; an application for which numerous excellent resources, toolboxes, and automated pipelines are readily available. Instead, as the Reviewer notes in their previous comment, the novelty of our contribution lies in the cluster-permutation statistics we present for circular data, exemplified here in the context of respiratory phase effects on behaviour and/or neural activity. Therefore, our stepwise tutorial takes care of accurate feature extraction (i.e., phase vectors), parameter selection (for phase binning), and computation of cluster-based group level statistics. This pipeline is explained in great detail on the corresponding GitHub repository with openly available software code provided in both MATLAB and Python as well as accompanying data.
As we state in the main text (kindly see l. 204), we refrain from detailing the pipeline at the level of single function commands since once the manuscript has been published, it cannot be updated following any further developments of the code itself. Rather, the step-by-step tutorial should be an openly available living document outside of the manuscript. Therefore, the step-by-step example is hosted independently on GitHub and frequently updated for all investigators to access the latest version of our code.

All that being said, we have revised both the main text as well as the GitHub repository in response to the Reviewer's comments. First, as suggested, we have rephrased Nexus #3 for a more detailed description of the workflow (l. 456):

> At this stage, we assume that respiratory phase has been extracted from the raw recordings (e.g. with two-point interpolation, Nexus 1), a surrogate distribution has been constructed using the IAAFT procedure (Nexus 2), and the outcome variable has been binned according to both empirical and surrogate phases. As a result, we have our outcome variable across one single vector of empirical and a large number (e.g. k = 5000) of surrogate phase vectors. [...] Recall that this procedure is executed for empirical and surrogate values, resulting in clusters for a single empirical distribution and a large number of surrogate distributions of outcome ~ phase bins.

Second, we have amended the openly accessible online material in the GitHub repository and now provide an additional markdown file describing a step-by-step workflow for all analyses mentioned in the paper, including references to the relevant code for each step. We have also added code for the computation of phase-amplitude coupling (as exemplified for the alpha band in Fig 3d) based on preprocessed M/EEG data. As previously mentioned, our tutorial was not intended to be focussed on the preprocessing of M/EEG data, thus we refer the Reviewer and all investigators to the tutorials and resources provided in the markdown file for detailed instructions on this preliminary step. The new additional resources are referenced in the main text as follows (l. 708):

> A step-by-step explanation including exemplary code for all analyses mentioned in this paper can be found in the “how_to_respmethods.md” file in the Github repository.
