# Supplementary material for: Robust circular cluster-based statistics for respiration-brain coupling
Source: PLoS Comput Biol. 2026 Sep 3;22(9):e1014672. doi: 10.1371/journal.pcbi.1014672 (PMC13541119; doi:10.1371/journal.pcbi.1014672)
Supplement: Supplementary file 10004 [file pcbi.1014672.s004.pdf]

Response to Reviewers

We would like to thank all reviewers for their positive feedback and constructive comments on our manuscript. All remaining minor suggestions are now included in a second round of revisions.
In what follows, we address all comments in a point-by-point reply. Reviewer comments are shown in **grey**, our responses in **blue**, and quoted changes to the manuscript in **red**.

Reviewer #1: I thank the authors for their careful and constructive revision. Overall, the response has improved the manuscript quite a bit, particularly through the addition validation steps, clearer framing of the circular cluster-based procedure, and more detailed guidance on the analysis workflow.

I've only a few remaining minor concerns (after reading the whole reply, namely, all reviewers' comments):

We thank the reviewer for their positive assessment of our revision and will respond to remaining minor points below.

1) If I've correctly understood the authors' notation, ω denotes the full width of the moving window, whereas Δφ denotes the distance between neighbouring bin centres. Given this notation, the condition for overlap should appear to be $\Delta\varphi < \omega$, not $\Delta\varphi < \omega/2$. Similarly, neighbouring windows would be contiguous when $\Delta\varphi = \omega$, and gaps would occur when $\Delta\varphi > \omega$. By contrast, if $\Delta\varphi = \omega/2$, the windows would still overlap by half of their width. Thus, unless I have misunderstood the intended definition of ω, the current description seems to compare Δφ with the half-width of the window rather than with the full window width. I suggest checking this.

We apologize for this oversight which has now been corrected (l. 287):

> Since the moving window is defined symmetrically around the center of each phase bin, it follows for each combination of k and ω that whenever $\Delta\varphi < \omega$, the incremental steps of the moving window are smaller than its width, which results in overlap. For $\Delta\varphi = \omega$, neighbouring phase bins would be exactly aligned, while $\Delta\varphi > \omega$ will likely lead to the adverse scenario that some trials are not considered in the analysis at all (since the moving window will not cover all phase angles).

2) The recommendation to select parameters using pilot or independent data is useful, but the criterion for selecting the final parameters remains somewhat underspecified. A short practical rule, for example based on minimum event counts per bin and stability of the phase-resolved estimate, would make the guidance more reproducible. Thanks

We appreciate the comment and understand the reviewer's insistence on more practical guidelines. As these are difficult to make (e.g. across studies with different expected effect sizes), the best recommendation we have for users is to treat phase bins as levels of an experimental factor and ensure that a sufficient number of events are included within each

phase bin (just like one would do in an event-related M/EEG study). We have added this piece of advice to the manuscript (l. 301):

> While it is difficult to provide definitive rules for a minimum number of events per phase bins, experimenters are generally well-advised to treat phase bins as factor levels and ensure that each bin contains a number of events similar to event-related analyses in M/EEG research, i.e. no fewer than 50 events (Borras et al., 2022).

3) The response to the issue of breath holds and plateau-like troughs is helpful, but I think the manuscript would still benefit from a more explicit rule for trough selection in ambiguous cases, for example whether the first minimum, last minimum, absolute minimum, a slope-based criterion, etc should be used.

Again, an explicit rule is difficult to provide and greatly depends on overall data quality as well as the respiratory recording device, but we have recommended either absolute minimum/maximum or the temporal centre of the plateau as general advice (l. 241):

> This detection can at times be ambiguous, e.g. in the case of transient breath holds (i.e. plateaus in the respiratory data). Here, we recommend assigning the peak/trough to the absolute maximum/minimum or the centre of the time frame for which the plateau is observed.

These points do not change my positive assessment of the revision, but addressing them would make the manuscript clearer and more robust.

Reviewer #2: Thank you for the detailed reply to my earlier comments, the article is in better shape now. I only have a few relatively minor comments at this stage.

It is great to see simulations, although i couldn't find the code on GitHub. It would be useful to make a table on the landing page, describing how to reproduce each figure from the article.

We apologize for this oversight - all code was online at the time of submission, but not on the main branch of the repository. In the meantime, the code has been consistently implemented on GitHub and we now also provide dedicated scripts to reproduce our figures in the folder matlab > _figures (figure 2 and 3) and python > Tutorial_PlotSimulation (simulation figures). The data visualized in the plots can be found in _exampledata (figure 2 and 3) and the accompanying, linked OSF project (full simulation results see: https://osf.io/yzewv/overview). All remaining figures are schematic diagrams created manually in Microsoft PowerPoint to aid conceptual understanding, and therefore do not have corresponding code.

I still find the various claims about "robustness" vague and in general inappropriate. To claim robustness when using t-tests on means, even when the T sampling distribution is obtained using a resampling method, is just statistically wrong. The mean is not robust, because a single value can dominate it. The same goes for the SD, making the T statistics not robust. In a different inferential context, the mean combined with bootstrap methods does not lead to robust inferences (Rousselet, Pernet & Wilcox, 2023). There are also well-known problems with permutation tests, in certain situations (Wilcox, 2022--chapter 5; Christensen & Zabriskie,

2022). And the standard permutation approach doesn't work to make inferences about interactions (Frossard & Renaud, 2021), so it is misleading to present the permutation framework as a general solution--it is a bit more complicated. Overall, making multiple claims about robustness without pointing out issues with permutation and inferences on means is misleading and a missed opportunity to educate the community. You wrote that the robustness claims are substantiated by the simulation results, but I couldn't find details about stress tests. I'm not saying that such simulations are required, simply that more careful descriptions are needed, with pointers to more robust solutions. This is important if your methods are applied to clinical populations in which case outliers are expected for instance.

Thank you for giving us the opportunity to clarify our use of the word ‘robust’ whose intended meaning is much more general than e.g. referring to any particular statistical parameter. We apologize for the ambiguity and hope that the revisions made in response to the reviewer’s remarks help improve the clarity of our manuscript.
By ‘robust circular cluster-based statistics’ in our title, we refer to the robustness of the entire pipeline up to and including the computation of inferential statistics. As we lay out in the paper, there are pitfalls and potentially bad choices in every step of the analysis pipeline, which is why we aimed to give practical advice, compare different methods for each analysis step, and now included simulations to verify the cluster correction in the last step. While we stand by (and provide evidence for) the claim that our pipeline increases overall robustness of analyses of respiration-brain coupling, we readily acknowledge that our use of the term in some instances is less warranted. Therefore, we have now clarified its intended meaning early in the manuscript (l. 56):

> Our aim is to provide a stepwise workflow which avoids common pitfalls for the benefit of overall robustness.

In addition, we have either rephrased or amended each instance of the term ‘robust’ throughout the manuscript (we marked changes only for clarity):

> This quest for valid methods is further complicated by the (naturally) circular data involved in the study of phase-locked effects, e.g. in respiration-brain coupling. (l. 21)
>
> Interpretation of effects is particularly affected by the problem of multiple comparisons in phase-related inferential statistics. (l. 25)
>
> This step concludes our example analysis and yields a valid cluster-based statistic to answer the initial research question, e.g. whether or not hit rate significantly fluctuates across the respiratory cycle (see Fig. 1c). (l. 199)
>
> Of note, interpolation approaches require a reliable detection of peaks and troughs in the raw respiratory trace. (l. 241)
>
> **Nexus 2: Adequate surrogate distributions using IAAFT** (l. 321)
>
> In addition to t-values as a measure of relative dispersion, we included a more robust measure based on the interquartile range and benchmarked our clustering approach

against the Omnibus correction for family-wise error rates [41] (but see Rousselet et al., 2023 for limitations). (l. 519)

We propose an easy-to-use, generalisable pipeline that can flexibly be implemented for any phase-locked analysis of neural, behavioural, or peripheral data. (l. 597)

In the tradition of 'Ten simple rules' papers, we close with synoptic recommendations which we hope will lead towards greater reliability, overall robustness, and reproducibility in brain-body neuroscience. (l. 602)

For now, the purpose of this tutorial was to provide an overview of a usable analytical pipeline particularly for inexperienced analysts in the rapidly growing field of brain-body neuroscience. (l. 710)

viii) Two possible measures of relative dispersion: t values and median/iqr, the latter being a measure used consistently for a more robust scaling against outliers [39, 40]. (l. 889)

We thank the reviewer again and hope that these clarifications help prevent the term from being overinterpreted in a statistical sense, which was never our intention.

About the group level cluster inference:

- It would be worth pointing out the finite-sample correction for p-values (Phipson & Smyth, 2010; Good, 2005), with p=(P(tobs>tperm)+1) / (k+1)

  The finite-sample correction has been added (l. 511):

  Note that for group-level cluster inference, additional correction methods such as finite-samples correction (Phipson & Smyth, 2010) can be implemented, if desired.

- The default seems to be a two-sided test, which doesn't afford directional claims. It would be more appropriate to offer one-sided tests by default (Rubin, 2022). This is directly related to your recommendation [1] in the discussion.

  Thank you - the alternative hypothesis is now a mandatory input (no default) to prevent users from inadvertently running the test with an unintended one-sided specification.

You do a great job describing issues with the interpretations of cluster-based inferences. However, in your suggested statement "Non-parametric cluster-based permutation testing indicated systematic modulation of hit rate across respiratory phase ($p < .05$)...", the term "systematic" is not warranted. A significant group effect is no guarantee that all participants show the group effect. A very careful phrasing would go something like that: "suggested that there is an effect of unknown sign, somewhere, for an unknown number of participants".

Thank you, the statement has been rephrased (l. 702):

> Rather, the analyst should report this finding in a manner that is less phase-specific, for instance: ‘Non-parametric cluster-based permutation testing indicated that there is a group-level effect of respiratory phase on hit rate (*p* < .05) with unknown sign, corresponding to a cluster found during the inspiratory phase’.

Another limitation worth pointing out, is that the effect localisation from group inferences has no uncertainty. A hierarchical sampling approach can be used to provide confidence intervals about onsets and offsets of effects for instance (Rousselet, 2025).

The limitation has been acknowledged and the reference has been added (l. 705):

> Note that confidence intervals for onsets and offsets of effects can be gained from a hierarchical sampling approach (Rousselet, 2025).

"to reliably control error rates" -> "to control error rates". Error rates are only define in the long-run, reliability is part of the definition, unless you mean something else.

The word ‘reliably’ has been removed (l. 600).

"Shuffling single time points is not a robust permutation approach" -- replace robust with valid or something more specific.

Since this refers to the general remarks about robustness (see above), this instance too has been rephrased (l. 649).

"Given the demonstrably strong influence of methodological choices..." In this important section of the discussion, it is worth pointing out the issue with experimenters' degrees of freedom and the need to pre-register analyses, or to provide sensitivity analyses over a range of plausible parameter values. Brain imaging is rife with p-hacking and HARKing.

The reviewer’s suggestion has been added to the Discussion section which now reads as follows:

> In the interest of reproducible science, these choices can be made a priori in a preregistered analysis protocol and/or the analyst provides sensitivity analyses across a range of sensible parameter values. (l. 671)

Reviewer #3: I thank the authors for their detailed response and for the substantial revisions made to the manuscript. The revised version is improved, and several of my previous concerns have been addressed at least in part. In particular, the authors now more clearly identify the main novelty of the work as a circular extension of cluster-based permutation testing, embedded within a practical pipeline for respiration phase-resolved analyses. The additional simulation-based validation also strengthens the manuscript and provides useful evidence regarding Type I error control and statistical power.

Thank you, we appreciate the reviewer’s positive assessment of our revision.

However, I do not find all responses fully satisfactory. In several cases, the authors have clarified the scope of the paper rather than directly addressing the conceptual and methodological concerns raised in the previous review. This is acceptable to some extent, but it should be made explicit in the manuscript so that the contribution is not overstated.

A central issue concerns the conceptual framing. The manuscript motivates the work within brain–body neuroscience, interoception, and organ-system interactions. However, in their response, the authors state that the manuscript does not aim to discuss respiration–brain coupling within a broader conceptualisation of physiology, because this has been done in previous work. I understand the wish to keep the present manuscript methodologically focused, but this response only partially addresses my concern. If the paper uses the broader brain–body and physiological-systems literature as its motivation, then the manuscript should more explicitly delimit what it does and does not contribute to that literature. Otherwise, there is a risk that the paper draws rhetorical strength from a broad brain–body framing while offering a much narrower methodological contribution.
Relatedly, the novelty should remain carefully framed. In my view, the novelty is not respiration–brain coupling itself, nor the use of respiratory phase, surrogate data, or cluster-based permutation testing per se. The genuinely novel element appears to be the adaptation of cluster-based permutation correction to circular phase-binned data, together with its integration into a tutorial workflow. This is a useful methodological contribution, but it is a relatively specific one. I therefore encourage the authors to ensure that the abstract, introduction, and discussion consistently present the work as a focused statistical/tutorial contribution rather than as a broader conceptual framework for brain–body physiology.

In response to the reviewer's comments in the first round, we have clearly outlined both our manuscript's contribution to the field of brain-body neuroscience (and beyond), as well as its key novelty, namely establishing a cluster-correction approach for circular data. In the previous round of comments, the reviewer identified exactly this novelty as the key contribution, and does so once again in their comments above. Following their initial suggestion to clearly outline our contributions in all major sections of the paper, we revised our manuscript accordingly. Among other changes, this included statements clearly distinguishing our practical tutorial pipeline from conceptual work:

> In this tutorial, we propose a robust pipeline for respiration phase-related analyses based on a novel circular extension of cluster-based permutation testing. We highlight and offer guidance on critical parameters in the analysis, systematically compare various approaches being used in the field today, and provide open-access software code for flexible use and future development of our proposed pipeline. (Abstract, l. 26)

> Therefore, in what follows, we describe an easy-to-use pipeline for continuous phase extraction and the joint analysis of respiratory and neural or behavioural data and introduce a novel extension of widely used cluster permutation testing for circular data. (Introduction, l. 114)

> The basic premise of this tutorial are research questions related to systematic covariation between the respiratory rhythm and behavioural or neural outcome variables: *Does hit rate in a perception task fluctuate over the respiratory cycle? What is the relationship between alpha power and respiration phase?* (Methods, l. 128)

In this tutorial paper, we present a unified methodological approach for phase-coupling analyses tailored to (but not limited to) applications in the field of respiration-brain interactions. We propose a robust and generalisable pipeline that can flexibly be implemented for any phase-locked analysis of neural, behavioural, or peripheral data. Our approach advances the status quo of brain-body research in two central aspects: First, we implement a unified way of generating surrogate phase distributions for statistical analysis. Second, we present a novel extension of cluster permutation procedures specifically for circular data in order to reliably control error rates in phase-related analyses. (Discussion, l 578)

As for the specificity argument, we fully acknowledged what our manuscript can and cannot provide in the initial submission already, but even more clearly after the first round of revision:

We want to emphasize that some aspects of our proposed pipeline generalize well to new applications whereas others are relatively specific to respiration-brain coupling. As outlined above, the cluster-based permutation approach extends well beyond any particular research question and as such is not limited to respiration phase-related analyses. Rather, it provides a general statistical framework for the analysis of behavioural or neural data in relation to any circular signal (e.g. recorded across respiratory phase, visual angles, or time of day) based on established practices in the field of neuroscience. As we note at the outset, however, the properties of physiological signals recorded from the heart, lungs, and stomach, are very diverse with regard to their respective frequencies. As a consequence, the systematic comparison of phase extraction methods was conducted specifically for respiratory analyses and will not hold for cardiac data (which are discretised into diastole and systole) or gastric data (which can usually be handled with a simple Hilbert transform). Moreover, the proposed IAAFT-based approach will likely not be beneficial for gastric recordings, for example: The low frequency combined with high regularity of the signal means that the IAAFT algorithm will have a hard time generating adequate surrogates whose phase is not highly correlated with that of the original signal. In practice, this means that after preprocessing of the gastric signal, another approach should be chosen to generate surrogate data for subsequent statistical comparisons. If the analyst then wants to quantify whether behavioural, neural, or other physiological outcomes show meaningful variability across the gastric cycle, they can easily integrate their externally generated surrogate vectors in our provided code for cluster-based permutation testing. (Discussion, l. 653)

‘Drawing rhetorical strength’ from any particular framing is quite precisely the opposite of how we lay out the limits of our contribution. The word ‘conceptual’ was used once referring to our approach and has now been rephrased:

Hence, while this paper lays out the practical framework of our proposed pipeline, we kindly refer the reader to the GitHub repository and its documentation for in-depth explanations of each function and practical example application scripts (l. 205).

In our view, the practical focus of this paper is unmistakably distinct from theoretical or conceptual frameworks of brain-body interactions. If they disagree, we would kindly ask the reviewer to provide specific examples of instances where they feel as if we were presenting

our hands-on statistical/methodological tutorial as anything beyond that. We are keen to make sure that our manuscript cannot be misconstrued as something it is not, have written the manuscript to the best of our abilities in this regard, and invite the reviewer to point out instances in which these efforts could be further improved.

The authors have also improved the discussion of why the manuscript focuses on respiratory phase rather than other respiratory descriptors. I appreciate that they now acknowledge the relevance of respiratory depth, pathway, rate, and variability. However, I still find the response somewhat oversimplified. The statement that these other respiratory features can generally be handled with straightforward statistical comparisons or linear models may be true in some experimental designs, but amplitude, rate, variability, and their interactions with neural signals can also pose non-trivial modelling challenges, especially in continuous, multimodal, and non-stationary recordings. I recommend softening this wording to avoid implying that non-phase respiratory descriptors are methodologically simple or secondary.

Thank you, this was by no means our intention and the wording has been rephrased as suggested (l. 44):

> Analyses of physiological parameters like breathing depth [7,8], pathways [9,10], or respiratory rate [11,12] can be conducted with straightforward statistical comparisons across two or more conditions (although exceptions certainly apply, e.g. in continuous or non-stationary recordings).

A further remaining concern concerns tutorial usability. The authors state that the step-by-step workflow is described in the GitHub repository rather than in detail in the manuscript because the code may evolve over time. While I understand this reasoning, a methods/tutorial paper should still be sufficiently self-contained for readers to understand the full analysis logic without relying entirely on an external living document. The revised Nexus 3 section is clearer, but the manuscript would benefit from a concise table, algorithmic checklist, or schematic workflow that explicitly links each analysis stage to its input and output: respiratory signal preprocessing, phase extraction, surrogate generation, phase binning, computation of outcome-by-phase matrices, group-level summary statistics, and circular cluster correction.

The suggested order of processing steps precisely reflect the order of our manuscript and is the reason we structured it that way. In order to make it easier for the reader to follow that workflow, we now explicitly mention it early on and implemented the reviewer's suggestion of a schematic workflow (l. 121):

> The main text of this tutorial follows a typical workflow going step by step from raw data inference statistics (Fig. 1): Raw respiratory data are preprocessed to yield respiratory phase information. Based on this phase extraction, we then create a large number of surrogate respiratory phase vectors. Using the empirical and surrogate phase vectors, experimental outcomes of interest are assigned to (surrogate) respiratory phase bins, resulting in lower-dimensional matrices of outcome-by-phase bin. On the group level, these individual matrices are used for summary statistics and we finally apply circular cluster-based correction for multiple comparisons.

Although the authors provide code, it appears that some of the data required to run the tutorial workflow are not included or not clearly accessible. In particular, the scripts seem to require

external files such as raw respiration recordings, an events file, and/or precomputed phase-binned empirical and surrogate matrices. For this reason, I was not able to fully evaluate what the code does in practice or to test whether the tutorial pipeline can be executed from beginning to end.

We thank the reviewer for raising this important point regarding accessibility. We fully agree that the tutorial workflow should be executable from beginning to end without requiring external files that were not provided. To address this, we have now added example data for all tutorial scripts to the repository, located in the _exampledata folder. This includes a raw respiration recording, the corresponding events file, and the precomputed phase-binned empirical and surrogate matrices required to run the event-based pipeline as well as an example dataset of concurrent neural and respiratory recordings for the continuous statistics tutorial. With these files in place, the reviewer (and future users) should be able to execute the complete tutorial workflow end-to-end and evaluate what each script does in practice.
