# Supplementary material for: Robust circular cluster-based statistics for respiration-brain coupling
Source: PLoS Comput Biol. 2026 Sep 3;22(9):e1014672. doi: 10.1371/journal.pcbi.1014672 (PMC13541119; doi:10.1371/journal.pcbi.1014672)
Supplement: Peer review history

# Peer review: Decision Letter 0

**DOI:** 10.1371/journal.pcbi.1014672.r001

**Authors:** Daniele Marinazzo

12 Feb 2026

PCOMPBIOL-D-26-00082

Robust circular cluster-based statistics for respiration-brain coupling

PLOS Computational Biology

Dear Dr. Kluger,

Thank you for submitting your manuscript to PLOS Computational Biology. After careful consideration, we feel that it has merit but does not fully meet PLOS Computational Biology's publication criteria as it currently stands. Therefore, we invite you to submit a revised version of the manuscript that addresses the points raised during the review process.

Please submit your revised manuscript by Apr 14 2026 11:59PM. If you will need more time than this to complete your revisions, please reply to this message or contact the journal office at ploscompbiol@plos.org. When you're ready to submit your revision, log on to https://www.editorialmanager.com/pcompbiol/ and select the 'Submissions Needing Revision' folder to locate your manuscript file.

Please include the following items when submitting your revised manuscript:

* A letter that responds to each point raised by the editor and reviewer(s). You should upload this letter as a separate file labeled 'Response to Reviewers'. This file does not need to include responses to formatting updates and technical items listed in the 'Journal Requirements' section below.

* A marked-up copy of your manuscript that highlights changes made to the original version. You should upload this as a separate file labeled 'Revised Manuscript with Track Changes'.

* An unmarked version of your revised paper without tracked changes. You should upload this as a separate file labeled 'Manuscript'.

If you would like to make changes to your financial disclosure, competing interests statement, or data availability statement, please make these updates within the submission form at the time of resubmission. Guidelines for resubmitting your figure files are available below the reviewer comments at the end of this letter

We look forward to receiving your revised manuscript.

Kind regards,

Daniele Marinazzo

Section Editor

PLOS Computational Biology

**Journal Requirements:**

If the reviewer comments include a recommendation to cite specific previously published works, please review and evaluate these publications to determine whether they are relevant and should be cited. There is no requirement to cite these works unless the editor has indicated otherwise.

1) We ask that a manuscript source file is provided at Revision. Please upload your manuscript file as a .doc, .docx, .rtf or .tex. If you are providing a .tex file, please upload it under the item type u2018LaTeX Source Fileu2019 and leave your .pdf version as the item type u2018Manuscriptu2019.

2) Please provide an Author Summary. This should appear in your manuscript between the Abstract (if applicable) and the Introduction, and should be 150-200 words long. The aim should be to make your findings accessible to a wide audience that includes both scientists and non-scientists. Sample summaries can be found on our website under Submission Guidelines:

https://journals.plos.org/ploscompbiol/s/submission-guidelines#loc-parts-of-a-submission

3) Your manuscript is missing the following sections: Introduction, Results, and Discussion.  Please ensure all required sections are present and in the correct order. Make sure section heading levels are clearly indicated in the manuscript text, and limit sub-sections to 3 heading levels. An outline of the required sections can be consulted in our submission guidelines here:

https://journals.plos.org/ploscompbiol/s/submission-guidelines#loc-parts-of-a-submission

4) Please upload all main figures as separate Figure files in .tif or .eps format. For more information about how to convert and format your figure files please see our guidelines:

https://journals.plos.org/ploscompbiol/s/figures

5) Some material included in your submission may be copyrighted. According to PLOSu2019s copyright policy, authors who use figures or other material (e.g., graphics, clipart, maps) from another author or copyright holder must demonstrate or obtain permission to publish this material under the Creative Commons Attribution 4.0 International (CC BY 4.0) License used by PLOS journals. Please closely review the details of PLOSu2019s copyright requirements here: PLOS Licenses and Copyright. If you need to request permissions from a copyright holder, you may use PLOS's Copyright Content Permission form.

Please respond directly to this email and provide any known details concerning your material's license terms and permissions required for reuse, even if you have not yet obtained copyright permissions or are unsure of your material's copyright compatibility. Once you have responded and addressed all other outstanding technical requirements, you may resubmit your manuscript within Editorial Manager.

Potential Copyright Issues:

i) Figure 1. Please confirm whether you drew the images / clip-art within the figure panels by hand. If you did not draw the images, please provide (a) a link to the source of the images or icons and their license / terms of use; or (b) written permission from the copyright holder to publish the images or icons under our CC BY 4.0 license. Alternatively, you may replace the images with open source alternatives. See these open source resources you may use to replace images / clip-art:

- https://commons.wikimedia.org

- https://openclipart.org/.

6) Please amend your detailed Financial Disclosure statement. This is published with the article. It must therefore be completed in full sentences and contain the exact wording you wish to be published.

1) State the initials, alongside each funding source, of each author to receive each grant. For example: "This work was supported by the National Institutes of Health (####### to AM; ###### to CJ) and the National Science Foundation (###### to AM)."

2) State what role the funders took in the study. If the funders had no role in your study, please state: "The funders had no role in study design, data collection and analysis, decision to publish, or preparation of the manuscript."

3) If any authors received a salary from any of your funders, please state which authors and which funders..

If you did not receive any funding for this study, please simply state: u201cThe authors received no specific funding for this work.u201d

**Reviewers' comments:**

Reviewer's Responses to Questions

**Comments to the Authors:**

**Please note here if the review is uploaded as an attachment.**

Reviewer #1: This manuscript presents a methodological tutorial and code-supported workflow for respiration phase analyses and respiration–brain coupling. The pipeline combines (i) respiratory phase extraction, emphasising landmark-based interpolation, (ii) surrogate generation, with a focus on iterated amplitude-adjusted Fourier transform (IAAFT) surrogates, and (iii) circular cluster-based permutation testing to address multiple comparisons while respecting wrap-around adjacency at phase boundaries. The authors aim to improve reproducibility and inferential validity in the respiration phase coupling literature.

The contribution is timely and likely to be useful for researchers working on respiration/body/brain interactions. The manuscript is well motivated and practically oriented. However, some elements would benefit from clearer formalisation, additional validation or methodological detail, and more explicit decision rules, particularly where analytical degrees of freedom may affect outcomes. The main points are as follows.

Major comments

1. Clarify the binning and windowing parameterisation (k, ω, overlap, coverage): The binning scheme is central to the workflow and to the claim that parameter choices influence sensitivity and power. However, the mapping between the number of bins (k), bin width (ω), overlap, and full-cycle coverage is not sufficiently explicit. In particular, small k settings are difficult to interpret when ω is fixed. It would be helpful to provide a concise formal specification of the scheme, including: (i) how bin centres are placed as a function of k, (ii) whether each bin is a sliding window of width ω centred on each bin centre, (iii) how overlap is defined, and (iv) how wrap-around membership is handled near ±π. A short worked example for small k would likely resolve ambiguity.

2. Specify a bit more a reproducible protocol for parameter tuning on an independent subset: The manuscript recommends selecting binning parameters on an independent subset to mitigate overfitting and improve power, but the practical procedure and selection criterion are underspecified. A simple split-sample or cross-validation protocol, paired with an objective criterion, would reduce subjectivity. For example, parameters could be chosen to stabilise an a priori summary metric while maintaining a minimum number of events per bin per participant.

3. Phase definition under breath holds and plateaus requires more explicit, physiology-informed rules: The tutorial motivates landmark-based interpolation using local maxima and minima, yet transition detection can be ambiguous in belt-based respiration (and other modalities) when breath holds or plateau-like segments occur near the expiratory trough. Choosing an earlier versus later minimum can extend one phase at the expense of the other. This matters because events near boundaries may be reassigned to different phases, potentially affecting estimates of phase locking and phase-resolved effects.

Relatedly, onset-focused alternatives, including trapezium-area approaches (Esra al.; Galvez-Pol et al. ; Engelen et al...) and slope or derivative-based onset detection, may better capture physiologically plausible transitions in some contexts. A physiology-informed choice would ideally yield phase durations consistent with typical breathing dynamics, rather than allowing signal artefacts to systematically stretch or compress phases across participants or tasks. I recommend (i) stating an explicit rule for trough selection under plateaus (for example, first versus last candidate minimum, or a slope-threshold criterion), (ii) justifying this choice with respect to respiratory physiology and measurement modality, and (iii) including a brief sensitivity analysis comparing an extremum-based approach with an onset-based alternative.

4. Provide additional validation of the circular cluster-based procedure: Readers may expect explicit evidence that the proposed procedure controls family-wise error under realistic null scenarios and has adequate power across effect widths and noise structures. A concise simulation-based validation, in the main text or supplement, varying sample size, effect width, event non-uniformity across phase, and noise characteristics, would strengthen confidence. A benchmark comparison to a simpler correction strategy (for example, max-statistic across bins) would further contextualise the method.

5. The manuscript notes differences between respiration and other rhythms, but it would be useful to delineate which components generalise beyond respiration. A short scope statement could distinguish: (i) general circular inference tools (for example, circular cluster correction), (ii) respiration-optimised phase extraction guidance, and (iii) conditions under which alternative surrogate or phase-definition strategies are preferable.

Minor comments

i) Terminology: Use “bin”, “window”, and “overlap” consistently, and/or define “quasi-continuous” early and precisely.

ii) Implementation details: Report default IAAFT settings, convergence checks, and approximate computational costs for typical signal lengths.

iii) Reporting guidance: Provide a brief reporting template specifying the phase-definition method, surrogate type, and correction method to support standardised reporting across studies.

Reviewer #2: Signed review: Guillaume Rousselet

This is a very interesting and useful article. You make a lot of important points, which are well explained in the main text and well summarised at the end. I haven't tried the code but the GitHub repository is well organised and documented. There two main points that you should address, and a few minor points to consider about the language.

##Main points:

The main issue is the lack of validation. Could you provide a simulation that demonstrates false positive rates and power for a few realistic cases? All new methods must be supported by reproducible simulations. The examples and rationale you provide are compelling, but not sufficient.

The second main point is about accessibility: in all figures, please increase the contrast and use colour-blind friendly colour palettes. In figure 3b, there is too much empty space and the colours are superfluous.

Panels in Figure 1 are too small. In figure 1c bottom panel, please remove the little stars. P and t values do not measure effect sizes, importance or reliability. A horizontal line marking statistical significance is sufficient.

##Minor points:

Robust statistics are mentioned in the abstract but not explain, in particular in relation to the specific issues in the literature. In other words, robust to what?

What are the particular statistical issues in your field? The introduction does a good job at summarising the issues with the statistical treatment of various measurements. The abstract should summarise these issues to motivate the work explicitly.

In the introduction, there is a lot of important points about binning. It would be worth pointing out the broader literature about the negative consequences of dichotomisation of continuous variables -- see for instance:

MacCallum RC, Zhang S, Preacher KJ, Rucker DD. On the practice of dichotomization of quantitative variables. Psychol Methods. 2002 Mar;7(1):19-40. doi: 10.1037/1082-989x.7.1.19

The cost of dichotomising continuous variables

https://www.ncbi.nlm.nih.gov/pmc/articles/PMC1458573/

In addition to Maris & Oostenveld (2007) you should cite the more directly relevant Maris, E., Schoffelen, J.-M., & Fries, P. (2007). Nonparametric statistical testing of coherence differences. Journal of Neuroscience Methods, 163(1), 161–175. https://doi.org/10.1016/j.jneumeth.2007.02.011

Also explain how your work relates to their effort dealing with phase coherence. In that context, is your method of computation of surrogate distributions really a permutation test? Typically, permutation relies on exchangeability between independent conditions. Here it seems you are approaching the data differently. Would it be more appropriate to describe your method as random shuffling without using the term permutation?

"Conventional cluster-based permutation tests assuming a non-circular independent variable fail to account for the wrap-around continuity inherent to circular data." -- but this is precisely because standard permutation tests address a different question, involving two independent groups, the assumption of exchangeability, to test whether the two distributions have identical shapes. Your approach addresses a different question.

Why t-test on means, given that sample means and sample SD are not robust? Could be trimmed means if you want to achieve robustness against outliers and skewness.

"t-tests and ANOVAs or their respective non-parametrical equivalents" -- in general, non-parametric models, such as rank-based models, tests different hypotheses than ANOVAs. To avoid that issue, and since a t-test is an ANOVA, you could write something like "parametric or non-parametric models".

"Phase-amplitude coupling means that the phase, for example

that of the respiratory rhythm, influences the amplitude of neural activity" -- is the causal language appropriate here or could you rephrase in terms of association?

"chance level" is mentioned several times. Could you rephrase to avoid readers thinking wrongly that NHST can determine if an effect is due to chance? This is not what you wrote, but we're one step away from an incorrect and common interpretation. Something like "larger than the effect from a random partition of the data" in the case of a permutation test. See more careful descriptions in Maris et al. (2007).

"Reliable extraction of respiratory phase" -- is reliability, in the sense of test-retest reliability, the appropriate term here? This section seems to be about accuracy instead.

Reviewer #3: This manuscript presents a methodological and tutorial-style framework for studying respiration–brain coupling, focusing on (i) respiratory phase extraction, (ii) surrogate data generation (with emphasis on IAAFT methods), and (iii) a circular extension of cluster-based permutation statistics to address multiple comparisons in phase-binned analyses. The topic is timely and relevant for the growing interest in brain–body interactions and interoceptive neuroscience. The proposed circular cluster-based inference is potentially valuable.

However, the manuscript currently lacks clear conceptual positioning, explicit novelty delineation, and a fully transparent analysis pipeline. Several aspects are presented in an oversimplified manner, particularly regarding respiratory signal features. Overall, the work would benefit from substantial clarification and restructuring to strengthen both scientific rigor and tutorial usability.

I therefore recommend major revision.

1) The manuscript discusses respiration–brain coupling within “brain-body neuroscience” and interoception frameworks but does not explicitly situate the work within the established network physiology paradigm, which has already introduced a systems-level view of interacting organ networks [1]. Moreover, the authors do not clearly contrast their approach with classical multimodal pipelines commonly used in the literature—such as extracting EEG spectral content by frequency band and relating it to autonomic indices (e.g., sympathovagal balance derived from HRV metrics computed from ECG).

2) Several statements implicitly treat respiratory phase as the primary or exclusive meaningful descriptor of respiration–brain interactions. This is an oversimplification. There is substantial literature demonstrating the importance of respiratory amplitude, volume, and breath-by-breath variability, including methods extracting respiratory amplitude aligned to cardiac R-peaks and analyzing its variability.

3)The manuscript employs phase–amplitude coupling where respiratory phase modulates neural amplitude. However, the rationale for privileging phase over other respiratory descriptors is not sufficiently justified. it would be equally informative to study modulation driven by respiratory amplitude dynamics, which may capture different physiological mechanisms.

4) A significant portion of the manuscript reads as a methodological overview of known problems (e.g., surrogate limitations, multiple-comparison issues), but it is not always clear what constitutes the novel contribution of the present work. At present, the main innovation appears to be: the circular cluster-based permutation statistic and its integration into a recommended pipeline. However, this is not explicitly framed.

5) Despite the presence of a methods synopsis and schematic figure, the manuscript does not provide a sufficiently explicit step-by-step workflow. As a result, it remains unclear how a reader should proceed starting from raw EEG (or MEG) signals to final statistical inference. I recommend also to better explain the flow of the paragraph named "Nexus 3: A novel approach for circular cluster-based permutation testing"

references

[1]- Bashan, Amir, et al. "Network physiology reveals relations between network topology and physiological function." Nature communications 3.1 (2012): 702.

**********

**Have the authors made all data and (if applicable) computational code underlying the findings in their manuscript fully available?**

The PLOS Data policy requires authors to make all data and code underlying the findings described in their manuscript fully available without restriction, with rare exception (please refer to the Data Availability Statement in the manuscript PDF file). The data and code should be provided as part of the manuscript or its supporting information, or deposited to a public repository. For example, in addition to summary statistics, the data points behind means, medians and variance measures should be available. If there are restrictions on publicly sharing data or code —e.g. participant privacy or use of data from a third party—those must be specified.

Reviewer #1: Yes

Reviewer #2: Yes

Reviewer #3: Yes

**********

PLOS authors have the option to publish the peer review history of their article (what does this mean?). If published, this will include your full peer review and any attached files.

If you choose “no”, your identity will remain anonymous but your review may still be made public.

**Do you want your identity to be public for this peer review?**  For information about this choice, including consent withdrawal, please see our Privacy Policy.

Reviewer #1: **Yes:** Alejandro (Alex) Galvez-Pol

Reviewer #2: **Yes:** Guillaume Rousselet

Reviewer #3: No

**Figure resubmission:**

While revising your submission, we strongly recommend that you use PLOS’s NAAS tool (https://ngplosjournals.pagemajik.ai/artanalysis) to test your figure files. NAAS can convert your figure files to the TIFF file type and meet basic requirements (such as print size, resolution), or provide you with a report on issues that do not meet our requirements and that NAAS cannot fix.

After uploading your figures to PLOS’s NAAS tool - https://ngplosjournals.pagemajik.ai/artanalysis, NAAS will process the files provided and display the results in the "Uploaded Files" section of the page as the processing is complete. If the uploaded figures meet our requirements (or NAAS is able to fix the files to meet our requirements), the figure will be marked as "fixed" above. If NAAS is unable to fix the files, a red "failed" label will appear above. When NAAS has confirmed that the figure files meet our requirements, please download the file via the download option, and include these NAAS processed figure files when submitting your revised manuscript.

**Reproducibility:**

To enhance the reproducibility of your results, we recommend that authors of applicable studies deposit laboratory protocols in protocols.io, where a protocol can be assigned its own identifier (DOI) such that it can be cited independently in the future. Additionally, PLOS ONE offers an option to publish peer-reviewed clinical study protocols. Read more information on sharing protocols at https://plos.org/protocols?utm_medium=editorial-email&utm_source=authorletters&utm_campaign=protocols

# Author comment: Author response to Decision Letter 1

**DOI:** 10.1371/journal.pcbi.1014672.r002

11 May 2026

Attachment Submitted filename: Berther_Resp2Revs_R1_260409.pdf

# Peer review: Decision Letter 1

**DOI:** 10.1371/journal.pcbi.1014672.r003

**Authors:** Daniele Marinazzo

29 May 2026

PCOMPBIOL-D-26-00082R1

Robust circular cluster-based statistics for respiration-brain coupling

PLOS Computational Biology

Dear Dr. Kluger,

Thank you for submitting your manuscript to PLOS Computational Biology. After careful consideration, we feel that it has merit but does not fully meet PLOS Computational Biology's publication criteria as it currently stands. Therefore, we invite you to submit a revised version of the manuscript that addresses the points raised during the review process.

Please submit your revised manuscript by Jul 29 2026 11:59PM. If you will need more time than this to complete your revisions, please reply to this message or contact the journal office at ploscompbiol@plos.org. When you're ready to submit your revision, log on to https://www.editorialmanager.com/pcompbiol/ and select the 'Submissions Needing Revision' folder to locate your manuscript file.

Please include the following items when submitting your revised manuscript:

* A letter that responds to each point raised by the editor and reviewer(s). You should upload this letter as a separate file labeled 'Response to Reviewers'. This file does not need to include responses to formatting updates and technical items listed in the 'Journal Requirements' section below.

* A marked-up copy of your manuscript that highlights changes made to the original version. You should upload this as a separate file labeled 'Revised Manuscript with Track Changes'.

* An unmarked version of your revised paper without tracked changes. You should upload this as a separate file labeled 'Manuscript'.

If you would like to make changes to your financial disclosure, competing interests statement, or data availability statement, please make these updates within the submission form at the time of resubmission. Guidelines for resubmitting your figure files are available below the reviewer comments at the end of this letter.

As the corresponding author, your ORCID iD is verified in the submission system and will appear in the published article. PLOS supports the use of ORCID, and we encourage all coauthors to register for an ORCID iD and use it as well. Please encourage your coauthors to verify their ORCID iD within the submission system before final acceptance, as unverified ORCID iDs will not appear in the published article. Only the individual author can complete the verification step; PLOS staff cannot verify ORCID iDs on behalf of authors.

We look forward to receiving your revised manuscript.

Kind regards,

Daniele Marinazzo

Section Editor

PLOS Computational Biology

Daniele Marinazzo

Section Editor

PLOS Computational Biology

**Reviewers' comments:**

Reviewer's Responses to Questions

**Comments to the Authors:**

**Please note here if the review is uploaded as an attachment.**

Reviewer #1: I thank the authors for their careful and constructive revision. Overall, the response has improved the manuscript quite a bit, particularly through the addition validation steps, clearer framing of the circular cluster-based procedure, and more detailed guidance on the analysis workflow.

I've only a few remaining minor concerns (after reading the whole reply, namely, all reviewers' comments):

1) If I've correctly understood the authors’ notation, ω denotes the full width of the moving window, whereas Δϕ denotes the distance between neighbouring bin centres. Given this notation, the condition for overlap should appear to be Δϕ < ω, not Δϕ < ω/2. Similarly, neighbouring windows would be contiguous when Δϕ = ω, and gaps would occur when Δϕ > ω. By contrast, if Δϕ = ω/2, the windows would still overlap by half of their width. Thus, unless I have misunderstood the intended definition of ω, the current description seems to compare Δϕ with the half-width of the window rather than with the full window width. I suggest checking this.

2) The recommendation to select parameters using pilot or independent data is useful, but the criterion for selecting the final parameters remains somewhat underspecified. A short practical rule, for example based on minimum event counts per bin and stability of the phase-resolved estimate, would make the guidance more reproducible. Thanks

3) The response to the issue of breath holds and plateau-like troughs is helpful, but I think the manuscript would still benefit from a more explicit rule for trough selection in ambiguous cases, for example whether the first minimum, last minimum, absolute minimum, a slope-based criterion, etc should be used.

These points do not change my positive assessment of the revision, but addressing them would make the manuscript clearer and more robust.

Reviewer #2: Thank you for the detailed reply to my earlier comments, the article is in better shape now. I only have a few relatively minor comments at this stage.

It is great to see simulations, although i couldn't find the code on GitHub. It would be useful to make a table on the landing page, describing how to reproduce each figure from the article.

I still find the various claims about "robustness" vague and in general inappropriate. To claim robustness when using t-tests on means, even when the T sampling distribution is obtained using a resampling method, is just statistically wrong. The mean is not robust, because a single value can dominate it. The same goes for the SD, making the T statistics not robust. In a different inferential context, the mean combined with bootstrap methods does not lead to robust inferences (Rousselet, Pernet & Wilcox, 2023). There are also well-known problems with permutation tests, in certain situations (Wilcox, 2022--chapter 5; Christensen & Zabriskie, 2022). And the standard permutation approach doesn't work to make inferences about interactions (Frossard & Renaud, 2021), so it is misleading to present the permutation framework as a general solution--it is a bit more complicated. Overall, making multiple claims about robustness without pointing out issues with permutation and inferences on means is misleading and a missed opportunity to educate the community. You wrote that the robustness claims are substantiated by the simulation results, but I couldn't find details about stress tests. I'm not saying that such simulations are required, simply that more careful descriptions are needed, with pointers to more robust solutions. This is important if your methods are applied to clinical populations in which case outliers are expected for instance.

About the group level cluster inference:

- It would be worth pointing out the finite-sample correction for p-values (Phipson & Smyth, 2010; Good, 2005), with p=(P(tobs>tperm)+1) / (k+1)

- The default seems to be a two-sided test, which doesn't afford directional claims. It would be more appropriate to offer one-sided tests by default (Rubin, 2022). This is directly related to your recommendation [1] in the discussion.

You do a great job describing issues with the interpretations of cluster-based inferences. However, in your suggested statement "Non-parametric cluster-based permutation testing indicated systematic modulation of hit rate across respiratory phase (p < .05)...", the term "systematic" is not warranted. A significant group effect is no guarantee that all participants show the group effect. A very careful phrasing would go something like that: "suggested that there is an effect of unknown sign, somewhere, for an unknown number of participants".

Another limitation worth pointing out, is that the effect localisation from group inferences has no uncertainty. A hierarchical sampling approach can be used to provide confidence intervals about onsets and offsets of effects for instance (Rousselet, 2025).

"to reliably control error rates" -> "to control error rates". Error rates are only define in the long-run, reliability is part of the definition, unless you mean something else.

"Shuffling single time points is not a robust permutation approach" -- replace robust with valid or something more specific.

"Given the demonstrably strong influence of methodological choices..."

In this important section of the discussion, it is worth pointing out the issue with experimenters' degrees of freedom and the need to pre-register analyses, or to provide sensitivity analyses over a range of plausible parameter values. Brain imaging is rife with p-hacking and HARKing.

###References

Christensen, William F., and Brinley N. Zabriskie. ‘When Your Permutation Test Is Doomed to Fail’. The American Statistician 76, no. 1 (2022): 53–63. https://doi.org/10.1080/00031305.2021.1902856.

Frossard, Jaromil, and Olivier Renaud. ‘Permutation Tests for Regression, ANOVA, and Comparison of Signals: The Permuco Package’. Journal of Statistical Software 99 (October 2021): 1–32. https://doi.org/10.18637/jss.v099.i15.

Good, Phillip I. Permutation, Parametric, and Bootstrap Tests of Hypotheses. 3rd edition. Springer, 2005.

Phipson, Belinda, and Gordon K. Smyth. ‘Permutation P-Values Should Never Be Zero: Calculating Exact P-Values When Permutations Are Randomly Drawn’. Statistical Applications in Genetics and Molecular Biology 9, no. 1 (2010). https://doi.org/10.2202/1544-6115.1585.

Rousselet, Guillaume A. ‘Using Cluster-Based Permutation Tests to Estimate MEG/EEG Onsets: How Bad Is It?’ European Journal of Neuroscience 61, no. 1 (2025): e16618. https://doi.org/10.1111/ejn.16618.

Rousselet, Guillaume A., Cyril R. Pernet, and Rand R. Wilcox. ‘An Introduction to the Bootstrap: A Versatile Method to Make Inferences by Using Data-Driven Simulations.’ Meta-Psychology 7 (December 2023). https://doi.org/10.15626/MP.2019.2058.

Rubin, Mark. ‘That’s Not a Two-Sided Test! It’s Two One-Sided Tests!’ Significance 19, no. 2 (2022): 50–53. https://doi.org/10.1111/1740-9713.01619.

Wilcox, Rand R. Introduction to Robust Estimation and Hypothesis Testing. 5th edn. Statistical Modeling and Decision Science. Academic Press, 2022.

Reviewer #3: I thank the authors for their detailed response and for the substantial revisions made to the manuscript. The revised version is improved, and several of my previous concerns have been addressed at least in part. In particular, the authors now more clearly identify the main novelty of the work as a circular extension of cluster-based permutation testing, embedded within a practical pipeline for respiration phase-resolved analyses. The additional simulation-based validation also strengthens the manuscript and provides useful evidence regarding Type I error control and statistical power.

However, I do not find all responses fully satisfactory. In several cases, the authors have clarified the scope of the paper rather than directly addressing the conceptual and methodological concerns raised in the previous review. This is acceptable to some extent, but it should be made explicit in the manuscript so that the contribution is not overstated.

A central issue concerns the conceptual framing. The manuscript motivates the work within brain–body neuroscience, interoception, and organ-system interactions. However, in their response, the authors state that the manuscript does not aim to discuss respiration–brain coupling within a broader conceptualisation of physiology, because this has been done in previous work. I understand the wish to keep the present manuscript methodologically focused, but this response only partially addresses my concern. If the paper uses the broader brain–body and physiological-systems literature as its motivation, then the manuscript should more explicitly delimit what it does and does not contribute to that literature. Otherwise, there is a risk that the paper draws rhetorical strength from a broad brain–body framing while offering a much narrower methodological contribution.

Relatedly, the novelty should remain carefully framed. In my view, the novelty is not respiration–brain coupling itself, nor the use of respiratory phase, surrogate data, or cluster-based permutation testing per se. The genuinely novel element appears to be the adaptation of cluster-based permutation correction to circular phase-binned data, together with its integration into a tutorial workflow. This is a useful methodological contribution, but it is a relatively specific one. I therefore encourage the authors to ensure that the abstract, introduction, and discussion consistently present the work as a focused statistical/tutorial contribution rather than as a broader conceptual framework for brain–body physiology.

The authors have also improved the discussion of why the manuscript focuses on respiratory phase rather than other respiratory descriptors. I appreciate that they now acknowledge the relevance of respiratory depth, pathway, rate, and variability. However, I still find the response somewhat oversimplified. The statement that these other respiratory features can generally be handled with straightforward statistical comparisons or linear models may be true in some experimental designs, but amplitude, rate, variability, and their interactions with neural signals can also pose non-trivial modelling challenges, especially in continuous, multimodal, and non-stationary recordings. I recommend softening this wording to avoid implying that non-phase respiratory descriptors are methodologically simple or secondary.

A further remaining concern concerns tutorial usability. The authors state that the step-by-step workflow is described in the GitHub repository rather than in detail in the manuscript because the code may evolve over time. While I understand this reasoning, a methods/tutorial paper should still be sufficiently self-contained for readers to understand the full analysis logic without relying entirely on an external living document. The revised Nexus 3 section is clearer, but the manuscript would benefit from a concise table, algorithmic checklist, or schematic workflow that explicitly links each analysis stage to its input and output: respiratory signal preprocessing, phase extraction, surrogate generation, phase binning, computation of outcome-by-phase matrices, group-level summary statistics, and circular cluster correction.

Although the authors provide code, it appears that some of the data required to run the tutorial workflow are not included or not clearly accessible. In particular, the scripts seem to require external files such as raw respiration recordings, an events file, and/or precomputed phase-binned empirical and surrogate matrices. For this reason, I was not able to fully evaluate what the code does in practice or to test whether the tutorial pipeline can be executed from beginning to end.

**********

**Have the authors made all data and (if applicable) computational code underlying the findings in their manuscript fully available?**

The PLOS Data policy requires authors to make all data and code underlying the findings described in their manuscript fully available without restriction, with rare exception (please refer to the Data Availability Statement in the manuscript PDF file). The data and code should be provided as part of the manuscript or its supporting information, or deposited to a public repository. For example, in addition to summary statistics, the data points behind means, medians and variance measures should be available. If there are restrictions on publicly sharing data or code —e.g. participant privacy or use of data from a third party—those must be specified.

Reviewer #1: Yes

Reviewer #2: **No:** I cannot find the simulation code on GitHub

Reviewer #3: Yes

**********

PLOS authors have the option to publish the peer review history of their article (what does this mean?). If published, this will include your full peer review and any attached files.

If you choose “no”, your identity will remain anonymous but your review may still be made public.

**Do you want your identity to be public for this peer review?**  For information about this choice, including consent withdrawal, please see our Privacy Policy.

Reviewer #1: **Yes:** Alejandro Galvez-Pol

Reviewer #2: **Yes:** Guillaume Rousselet

Reviewer #3: No

[NOTE: If reviewer comments were submitted as an attachment file, they will be attached to this email and accessible via the submission site. Please log into your account, locate the manuscript record, and check for the action link "View Attachments". If this link does not appear, there are no attachment files.]

**Figure resubmission:**

While revising your submission, we strongly recommend that you use PLOS’s NAAS tool (https://ngplosjournals.pagemajik.ai/artanalysis) to test your figure files. NAAS can convert your figure files to the TIFF file type and meet basic requirements (such as print size, resolution), or provide you with a report on issues that do not meet our requirements and that NAAS cannot fix.

After uploading your figures to PLOS’s NAAS tool - https://ngplosjournals.pagemajik.ai/artanalysis, NAAS will process the files provided and display the results in the "Uploaded Files" section of the page as the processing is complete. If the uploaded figures meet our requirements (or NAAS is able to fix the files to meet our requirements), the figure will be marked as "fixed" above. If NAAS is unable to fix the files, a red "failed" label will appear above. When NAAS has confirmed that the figure files meet our requirements, please download the file via the download option, and include these NAAS processed figure files when submitting your revised manuscript.

**Reproducibility:**

To enhance the reproducibility of your results, we recommend that authors of applicable studies deposit laboratory protocols in protocols.io, where a protocol can be assigned its own identifier (DOI) such that it can be cited independently in the future. Additionally, PLOS ONE offers an option to publish peer-reviewed clinical study protocols. Read more information on sharing protocols at https://plos.org/protocols?utm_medium=editorial-email&utm_source=authorletters&utm_campaign=protocols

# Author comment: Author response to Decision Letter 2

**DOI:** 10.1371/journal.pcbi.1014672.r004

28 Jul 2026

Attachment Submitted filename: Bertheretal_R2_Resp2Refs_260727.pdf

# Editor report: Decision Letter 2

**DOI:** 10.1371/journal.pcbi.1014672.r005

**Authors:** Daniele Marinazzo

5 Aug 2026

Dear Prof. Kluger,

We are pleased to inform you that your manuscript 'Robust circular cluster-based statistics for respiration-brain coupling' has been provisionally accepted for publication in PLOS Computational Biology.

Before your manuscript can be formally accepted you will need to complete some formatting changes, which you will receive in a follow up email. A member of our team will be in touch with a set of requests.

Please note that your manuscript will not be scheduled for publication until you have made the required changes, so a swift response is appreciated.

IMPORTANT: The editorial review process is now complete. PLOS will only permit corrections to spelling, formatting or significant scientific errors from this point onwards. Requests for major changes, or any which affect the scientific understanding of your work, will cause delays to the publication date of your manuscript.

Should you, your institution's press office or the journal office choose to press release your paper, you will automatically be opted out of early publication. We ask that you notify us now if you or your institution is planning to press release the article. All press must be co-ordinated with PLOS.

Thank you again for supporting Open Access publishing; we are looking forward to publishing your work in PLOS Computational Biology.

Best regards,

Daniele Marinazzo

Section Editor

PLOS Computational Biology

Daniele Marinazzo

Section Editor

PLOS Computational Biology

***********************************************************

# Editor report: Acceptance letter

**DOI:** 10.1371/journal.pcbi.1014672.r006

**Authors:** Daniele Marinazzo

PCOMPBIOL-D-26-00082R2

Robust circular cluster-based statistics for respiration-brain coupling

Dear Dr Kluger,

I am pleased to inform you that your manuscript has been formally accepted for publication in PLOS Computational Biology. Your manuscript is now with our production department and you will be notified of the publication date in due course.

The corresponding author will soon be receiving a typeset proof for review, to ensure errors have not been introduced during production. Please review the PDF proof of your manuscript carefully, as this is the last chance to correct any errors. Please note that major changes, or those which affect the scientific understanding of the work, will likely cause delays to the publication date of your manuscript.

Soon after your final files are uploaded, unless you have opted out, the early version of your manuscript will be published online. The date of the early version will be your article's publication date. The final article will be published to the same URL, and all versions of the paper will be accessible to readers.

For Research, Software, and Methods articles, you will receive an invoice from PLOS for your publication fee after your manuscript has reached the completed accept phase. If you receive an email requesting payment before acceptance or for any other service, this may be a phishing scheme. Learn how to identify phishing emails and protect your accounts at https://explore.plos.org/phishing.

Thank you again for supporting PLOS Computational Biology and open-access publishing. We are looking forward to publishing your work!

With kind regards,

Kannan R K Kuppusamy, B.TECH BIOTECHNOLOGY

PLOS Computational Biology | Carlyle House, Carlyle Road, Cambridge CB4 3DN | United Kingdom ploscompbiol@plos.org | Phone +44 (0) 1223-442824 | ploscompbiol.org | @PLOSCompBiol
